# Migrating bubble synthesis promotes mutagenesis through lesions in its template

**DOI:** 10.1093/nar/gkac520

**Published:** 2022-06-24

**Authors:** Beth Osia, Jerzy Twarowski, Tyler Jackson, Kirill Lobachev, Liping Liu, Anna Malkova

**Affiliations:** Department of Biology, University of Iowa, Iowa City, IA 52245, USA; Department of Cancer Genetics and Epigenetics, City of Hope, Duarte, CA 91010, USA; Department of Biology, University of Iowa, Iowa City, IA 52245, USA; Department of Biology, University of Iowa, Iowa City, IA 52245, USA; Department of Molecular and Human Genetics, Baylor College of Medicine, Houston, TX 77030, USA; School of Biological Sciences, Georgia Institute of Technology, Atlanta, GE 30332, USA; Department of Biology, University of Iowa, Iowa City, IA 52245, USA; Department of Biology, University of Iowa, Iowa City, IA 52245, USA

## Abstract

Break-induced replication (BIR) proceeds via a migrating D-loop for hundreds of kilobases and is highly mutagenic. Previous studies identified long single-stranded (ss) nascent DNA that accumulates during leading strand synthesis to be a target for DNA damage and a primary source of BIR-induced mutagenesis. Here, we describe a new important source of mutagenic ssDNA formed during BIR: the ssDNA template for leading strand BIR synthesis formed during D-loop migration. Specifically, we demonstrate that this D-loop bottom template strand (D-BTS) is susceptible to APOBEC3A (A3A)-induced DNA lesions leading to mutations associated with BIR. Also, we demonstrate that BIR-associated ssDNA promotes an additional type of genetic instability: replication slippage between microhomologies stimulated by inverted DNA repeats. Based on our results we propose that these events are stimulated by both known sources of ssDNA formed during BIR, nascent DNA formed by leading strand synthesis, and the D-BTS that we describe here. Together we report a new source of mutagenesis during BIR that may also be shared by other homologous recombination pathways driven by D-loop repair synthesis.

## INTRODUCTION

For much of the cell cycle, DNA remains double-stranded. However, during some processes of DNA metabolism, single-stranded DNA (ssDNA) becomes transiently exposed. Examples of such processes include S-phase replication, homologous recombination, transcription and DNA resection at uncapped telomeres or double strand breaks (DSBs) (reviewed in (1)). While lesions in the context of double-stranded DNA (dsDNA) can often be excised and repaired using the opposite strand as a template (reviewed in (2)), exposed ssDNA is vulnerable to a variety of damages, and is more limited in its capacity for error-free repair of DNA lesions. Moreover, with the exception of direct reversal, repair of lesions in ssDNA often leads to mutations (1).

Hypermutability of ssDNA has been observed in the presence of damaging agents that preferentially target ssDNA. Such ssDNA damages include base-alterations inflicted by alkylating agents (e.g. methyl methanesulfonate (MMS)) (3–5), oxidative damage (6,7), and cytidine deamination induced by cytidine deaminases such as AID/APOBEC enzymes (5,8–15). Damages inflicted in ssDNA often lead to the formation of mutation clusters (groups of closely spaced mutations) that are formed in regions of exposed ssDNA (1,4,5,11,14,16–21). These mutation clusters are also often strand-coordinated (comprised of the same mutant base present in the same DNA strand) (1,4,5,8,17,22). Thus, mutation clusters observed in yeast model systems and in human cancers have served as markers of persistent ssDNA (1,4,5,8–10,12,14,16,18,22–25). In addition, the pattern of strand coordination in mutation clusters can inform on the mechanism of ssDNA formation. For example, ssDNA formed by bidirectional resection of a DSB usually leads to the formation of clusters with switching strand coordination (1,5,17,25), while ssDNA formed in the nascent strand of BIR often leads to non-switching coordinated mutation clusters (4,17,21).

Another important property of persistent ssDNA is its ability to stimulate formation of secondary (non-B) DNA structures. S-phase DNA replication often stalls at secondary DNA structures formed in the template, and this leads to genetic instabilities, including mutations and chromosomal rearrangements (26–30). For example, in humans, G-quadruplexes (G4) that form in ssDNA during transcription were shown to promote recombination in patients with Bloom syndrome, presumably due to stalling of replication at G4 structures (31). Extensive analysis of cancer genomes revealed significant association between sequences with the potential to form non-B DNA structures (Hairpin/cruciform, G4, triplex, etc.) and rearrangement breakpoints (26,28,32,33). Additionally, studies in yeast demonstrated that inverted DNA repeats (IR) that can adopt a hairpin (stem-and-loop) structure in ssDNA, stimulate deletions and genomic rearrangements (34–42). Hairpin structures may form in ssDNA exposed during lagging strand synthesis, promoting stalling of the replicative DNA polymerase at the base of the hairpin, and often leading to replication slippage that produces deletions (26,34,36,37). Replication slippage (based on results obtained in various organisms (26,36,37,43,44)) often proceeds between short repeats (microhomologies) that are brought into close proximity by formation of the hairpin by IRs. It was proposed that the frequency of such IR-induced polymerase slippage at positions of microhomology is directly proportional to the frequency of hairpin formation, which is tied to the length and persistence of ssDNA (26,34–37,45).

Aside from S-phase replication, some types of repair DNA synthesis promote accumulation of ssDNA (1,46–50). For example, repair of double-strand DNA breaks (DSBs) through a pathway called break-induced replication (BIR) is particularly susceptible to accumulation of ssDNA that is both longer and more persistent than the relatively short-lived ssDNA exposed during S-phase replication (4,21,51). BIR is initiated when only one DSB end can find homology in the genome for strand invasion, resulting in the formation of a D-loop structure that is typical of all homologous recombination (HR) (reviewed in (52–55)). Studies of BIR using the yeast *Saccharomyces cerevisiae* demonstrated that BIR, like other types of HR, is preceded by extensive 5’-to-3’ end resection, exposing a long single-stranded 3’ end that is then covered by RPA, and later by Rad51 protein to initiate strand invasion (56–60). BIR synthesis is initiated at the 3’invaded end and proceeds via a migrating D-loop where branch migration displaces the newly synthesized leading strand. Unlike S-phase DNA synthesis, BIR is asynchronous, and the leading nascent strand accumulates as a long track of ssDNA behind the migrating D-loop (51,61). Lagging strand synthesis follows and uses the leading strand as a template, which leads to conservative inheritance of newly synthesized DNA (51,62). The long track of ssDNA accumulated behind the BIR D-loop was shown to form long and dense mutation clusters when alkylating damage was present or APOBEC3A (A3A) was expressed during BIR (4,21). A3A specifically targets ssDNA, generating mutations in cytosines preferentially at TCA and TCT (together referred to as TCW) motifs by converting cytidine to deoxyuridine (dU) (10,24). The dU lesions produced by A3A, as well as by other types of APOBEC enzymes, are excised by the uracil-DNA glycosylase Ung1, producing abasic (AP) sites that can promote mutagenesis via translesion synthesis or can often be bypassed without generating mutations by “error-free” pathways that likely involve recombination or template switching (11,20–22,63). When Ung1 is absent, all dUs formed by APOBEC persist and promote formation of C to T mutations by incorporation of adenine across from dU (3,11). Thus, the most accurate measurement of the length of ssDNA accumulated during BIR is achieved by assessing the length of mutation clusters generated *in ung1Δ* mutants during BIR in the presence of A3A (21). These clusters can be formed by A3A lesions in ssDNA produced either by resection preceding BIR or by BIR synthesis. Yet, because both lead to accumulation of clusters of C to T mutations, the individual contributions of resection and synthesis to the total amount of persistent ssDNA during BIR have not yet been determined (21). It also remains unknown whether the long ssDNA track formed during BIR can promote the formation of other types of mutations typical to persistent ssDNA, such as deletions of quasi-palindromic sequences due to polymerase slippage. Additionally, other structures formed during BIR that can potentially serve as sources of ssDNA (e.g. the template strand exposed during D-loop migration), have not yet been assessed for their mutagenic propensity.

Here, using an inducible BIR system, we further investigated the ssDNA intermediates formed during BIR by exploiting their vulnerability to A3A-inflicted damage and by assessing their propensity for replication slippage between microhomologies promoted by IRs. We determined that IR sequence placed on the track of BIR undergoes frequent deletions at microhomologies that flank the IRs. We propose that these deletions are promoted by hairpins formed by IRs when they are included into ssDNA formed during BIR. As previously reported, leading strand BIR synthesis provides one source of ssDNA that accumulates behind the BIR bubble as a result of asynchrony between leading and lagging strand synthesis. Also, our data suggest another source of ssDNA that can promote IR-mediated polymerase slippage: the template for the leading strand inside the D-loop (D-loop bottom template strand) that we have termed here the D-BTS.

In addition, we examined the vulnerability of the D-BTS to A3A damage and determined that mutagenic ssDNA is formed within the D-BTS region along the entire track of BIR. In sum, through two experimental approaches, we have identified ssDNA within the BIR D-BTS region, where leading strand synthesis takes place, as a new potent source of mutagenesis.

## MATERIALS AND METHODS

### Yeast strain construction and growth conditions

The yeast strains used for all experiments in this study are isogenic derivatives of AM1003, which contains two copies of Chromosome III (Chr III): one copy (recipient) is truncated and contains a recognition site for HO endonuclease at the *MAT*a locus, where a DSB can be introduced following HO induction by addition of galactose. Another copy of chromosome III (the donor) is full-length and cannot be cut by HO due to *MATα-inc* mutation. For a complete list of all strains constructed for this study, and those used in this work that were published previously, see Supplementary Data S1. Construction of AM1003 is fully described in (64) and AM1003 has the following genotype: *hmlΔ*::*ADE1*/*hmlΔ*::*ADE3 MATa*-*LEU2*-*tel*/*MATα-inc hmrΔ*::*HPH FS2Δ*::*NAT*/*FS2 leu2*/*leu2*-*3,112 thr4 ura3-52 ade3*::*GAL*::*HO ade1 met13*

To construct the strains containing the *lys2-InsH* reporter at three different positions in the donor chromosome, we used three derivatives of AM1003 that contained *THR4* inserted at the *thr4* position of the *MAT*α-inc chromosome, and an insertion of *LYS2* at the *MAT*α (replacing *MAT*α-inc region starting from the position located 249bp centromere-proximal from the border of the X–Y regions of the *MAT* locus and finishing at the position 5bp centromere-proximal to the Yα-Z1 border), at 16 kb or at 36 kb positions (see (65) for details).

The strains with insertions of *LYS2* at *MAT* and 16 kb positions have been described previously (65), while the strain with *LYS2* at 36 kb has been constructed here by transformation of *THR4* AM1003 derivative ((64), see Supplementary Data S1) with a DNA fragment obtained by amplifying the *LYS2* gene from the pLL12 plasmid (66) using the following primers (5’ to 3’) where lower-case letters indicate homology to the wild-type *LYS2* gene sequence and capital letters indicate homology to the respective position on Chr III:

Forward primer (FP):

ATCGTAAATACATAGGCTGGGCCATATACACTAACATGTGTCGTGACCAATGTGCAGCAGATAGACTTGCTCATTAAAaattacataaaaaattccggcgg and

reverse primer (RP):

AACTGGAAATGCTTTCCCTTTTGCCCTATCATTATTTTCTTTCCGATGTTATGCTTATTATATCTGTGATTGATAAGAGAttaagctgctgcggagcttcc

To insert the *lys2-InsH* reporter at three positions in the donor chromosome, a *pCORE* construct (containing *KanMX* and *URA3* cassettes (67,68)) was inserted into the *LYS2* gene, and then replaced by *lys2-InsH* sequence described in (36). Construction of strains containing the *lys2-A_4_* reporter cassette is described in (65). Reporter strains were confirmed by PCR and phenotype at each step of construction. “No DSB” strains were created by plating on YEP-Gal media and selection of colonies with an alpha-mating, Ade^+^ Leu^+^ phenotype that results from gene conversion (GC) repair of the DSB at *MAT*a. Strains containing the *ura3-29* reporter marked by *HPH* (that were later replaced by *Bleo*^r^) at 16kb and 90kb positions were originally constructed in (21) and used here to construct strains expressing A3A and empty vector (EV) plasmids in *UNG1* and *ung1Δ* backgrounds (see Supplementary Data S1).

Yeast strains containing *rev3::BSD*, *rad30::KanMX*, *HPH::KanMX*, *KanMX::Bleo*^r^, *pol4::KanMX* or *ung1::BSD* disruptions were constructed by transforming the parent strain with a PCR-amplified blasticidin (BSD) marker (TEF/BSD from Invitrogen), *KanMX* marker (69) or phleomycin-resistant Bleo^r^ marker (70). Primers used for marker amplification contained tail sequences homologous to the first and last 80 bp of the open reading frame of each gene to be disrupted (69). Successful disruptions were confirmed by both PCR and by the phenotypes of transformants (where possible). Mutants harboring the *pol3-Y708A* and *pol3-t* mutations were constructed by restriction digest of plasmids p170 (71) and p171 (72) respectively with HpaI prior to transformation. The *pol3-01* mutant was created by co-transformation of the CRISPR-Cas9 expression vector bRA89 (73) modified to express sgRNAs targeting the sequences (5′-3′): TCCTTTGATATCGAGTGTGC and the following repair template (5′-3′):

CAGCTCCATTGCGTATCATGTCCTTTGCTATCGCGTGTGCTGGTAGGATTGGCGTCTTTCCGGAACCTGAATACGATCCC.

Synthetic dropout (Sc), rich growth media (YEPD – yeast extract, peptone, and dextrose), YEP-lactate (YEP-Lac) and YEP-galactose (YEP-Gal) media were prepared similar to (74). Expression of APOBEC3A was achieved by transformation with an A3A-expression vector (pSR355) or EV control (pSR419) plasmids containing an *HPH* marker (22). Selection for correct transformants was done similar to (21). Yeast cultures were grown at 30°C for most experiments. Yeast strains harboring the temperature sensitive *pol3-t* mutation were grown at 18°C prior to DSB induction.

### Determining the rate of BIR-associated mutagenesis

Yeast strains from single colonies were grown with agitation in Sc media lacking leucine for approximately 20 h, diluted 20× with YEP-Lac and grown to logarithmic phase (for ∼16 h). DSBs were induced by addition of galactose to a final concentration of 2% (w/v). Due to residual BIR, which is capable of affecting Lys^+^ frequency even prior to galactose addition (74), the level of Lys^+^ during S-phase replication was determined in no-DSB control strains where HO recognition sites have been removed (64,65).

“No DSB” control strains were grown under the same conditions. In experiments including A3A and empty vector expression, hygromycin (1% w/v) was added to the YEP-Lac medium. After DSB induction, cultures were incubated at 30°C (or 20°C for low-temperature experiments as specified in Supplementary Figure S3) for 7h with agitation.

Appropriate amounts of culture were plated at 0h (before galactose addition) and 7h (after galactose addition) time points on YEPD and Sc-lysine dropout media (Sc-Lys) (and on Sc-adenine dropout (Sc-Ade) and Sc-adenine/lysine dropout (Sc-Ade/Lys) media for *POL3* mutant experiments) for strains harboring the *lys2-InsH* reporter cassette, or Sc-Ade dropout and Sc-adenine/uracil dropout media (Sc-Ade/Ura) for strains harboring the *ura3-29* reporter cassette. In experiments using *lys2-InsH* reporter strains, DSBs were initiated, DSB repair outcomes were identified, BIR efficiencies were calculated, and frequencies and rates of Lys^+^ reversions were determined as described in (65,75). For *ura3-29* reporter strains containing A3A or empty vectors, experiments were performed, BIR repair outcomes were identified, BIR efficiencies were calculated, and Ura^+^ reversion rates were determined similar to (51). Rates and frequencies are reported as median values and 95% confidence intervals (or ranges for experiments with fewer than 6 biological replicates). Mann–Whitney *U* test was used to draw statistical comparisons of mutation rates between different strains. Fisher's exact test was used to make statistical comparisons between fractions of different categories of repair outcomes.

To analyze mutation spectra, Lys^+^ or Ura^+^ colonies were selected randomly from 7h plates from experiments where cells underwent BIR (all colonies were considered to be independent BIR events due to no additional cell divisions before plating and big increase of 7 h over 0 h mutation frequency). For “No DSB” controls, only one colony (Lys^+^) was selected from each independent culture after plating (similar to (65)). Lys^+^ mutation spectra were determined by PCR amplification of *LYS2* alleles using the following primers in Lys^+^ outcomes (5’ to 3’): CCATCCACTTCTCATCTGAAAGACC, and AAATGTCACTGCAAATTATGCGGAAGAC. The PCR products were then Sanger sequenced using the following primer (5’ to 3’): GTTCGTACCCCTCTCGAGAATA. Lys^+^ outcomes were confirmed to be heterozygous after completion of BIR using the following primer pair, where the forward primer anneals to the spacer region between the repeats of the InsH quasi-palindrome and is indicative of the presence of an unaltered *lys2-InsH* allele (Lys^−^), while the reverse primer anneals to the *LYS2* sequence outside of the quasi-palindrome (5’ to 3’): ATCCTGGAAAACGGGAAAGG and AAATGTCACTGCAAATTATGCGGAAGAC respectively. Outcomes that did not produce a product using these two primers were considered to be homozygous and excluded from spectra results. Likewise, Ura^+^ mutation spectra were determined by PCR amplification of Ura^+^ outcomes using the following primers (5’ to 3’): GTGTGCTTCATTGGATGTTCGTAC, and AAAAGGCCTCTAGGTTCCTTTGTT. The PCR products were then Sanger sequenced using the following primer (5’ to 3’): CTGGAGTTAGTTGAAGCATTAGG. Homozygous substitutions leading to Ura^+^ reversions that were detected by Sanger sequencing were excluded from spectra results. Fisher's exact test was used to make statistical comparisons of mutation spectra between different strains for both reporter systems.

### Determination of transformation efficiency for various Lys^+^*lys2-InsH* deletion outcomes

PCR-amplified products from Lys^+^ outcomes after BIR (wild-type *LYS2*, Type I and Type II imprecise deletions) were obtained using the following primers (5’ to 3’): GAGGGATCCAAATGTTATTTCAACTATCA, and AAATGTCACTGCAAATTATGCGGAAGAC. Cultures of strains (Lys^−^) containing a *lys2-A_4_* reporter cassette (65) at *MAT*, 16 kb and 36 kb positions (see Supplementary Data S1) were grown to saturation, and each reporter strain was transformed with 1.5 μg of one of the amplified Lys^+^*lys2-InsH* outcomes to replace the *lys2-A_4_* reporter allele. Transformation efficiencies were measured by the frequency of Lys^+^ transformants (colonies) per 1ml of culture transformed with 1.5 μg of DNA plated (cell concentration determined by plating serial dilutions on YEPD) for each strain and input DNA combination. No DNA control transformations were also performed to measure the frequency of spontaneous Lys^+^ reversions of the *lys2-A_4_* cassette.

### Analysis of strand-specific mutations by whole-genome sequencing

Data from 25 *UNG1* and 25 *ung1Δ* BIR outcomes from our previous study (21) as well as an additional 37 *UNG1* and 20 *ung1Δ* BIR outcomes, all containing a *ura3-29* reporter marked by *Bleo*^r^ marker at the 90 kb position in Ori2, were prepared and sequenced as described in (21). Mutect2 (https://gatk.broadinstitute.org/hc/en-us/articles/360037593851-Mutect2) was used to call variants against the AM1003 reference genome containing a *ura3-29* reporter at 90 kb position. Variants with allelic frequencies lower than 0.35 were removed. Homozygous mutations were determined by allelic frequency of 0.85 or higher. Variants with allelic frequencies lower than 0.85 were called heterozygous. Identical mutations that occurred at the same chromosomal positions in different biological replicates were classified as existing prior to BIR induction and were thus discarded. C to N variants along the BIR track were appropriated as markers of A3A-induced lesions in the ssDNA template for lagging strand synthesis. Likewise, G to N variants along the BIR track were appropriated as markers of A3A-induced cytidine deamination in ssDNA of the template for the leading strand synthesis (D-loop bottom template strand (D-BTS)). Only C to N and G to N mutations on the right arm of Chr III were counted as representing BIR-related A3A-induced mutations.

Simulations of randomly distributed G to N mutations were performed by identifying the cumulative number of G to N positions across all sequenced samples on all chromosomes (total of 98 mutations) and redistributing them randomly across a synthetic genome to generate 100 000 unique samplings. From each of these samplings, the number of G to N mutations that occurred on the right arm of Chr III was recorded and used to create a frequency histogram and kernel density estimation. All custom code used for the variant filtering, simulations and graphing is available through GitHub (https://github.com/malkovalab/WGS-A3A-Tools).

### Deep sequencing

Strains containing the Ori1 or Ori2 *lys2-InsH* reporter at the 16kb position (Supplementary Data S1) were used to perform BIR-induction experiments as described in (74). For DNA purification, 5 ml of cell cultures containing ∼3 × 10^7^cells/ml were collected before (0 h) galactose addition and 12 h after galactose addition, and genomic DNA was extracted using the glass bead protocol as described in (21). The region corresponding to the insertion of *insH* in *LYS2* (∼700 bp-long) was amplified by PCR from these samples by using the following primers:

Forward primer 1 (5’ to 3’): AAATGTCACTGCAAATTATGCGGAAGAC

Reverse primer 1 (5’ to 3’): TGATAGTTGAAATAACATTTGGATCCCTC

The PCR products were separated by gel electrophoresis (using 1% agarose). Following electrophoresis, PCR fragments of ∼400–700 bp were excised from the gel and used for gel extraction using QIAquick Gel Extraction Kit (QIAGEN #28704). The gel extracted products were subsequently used for a second PCR amplification of a ∼500 bp region using the following primers:

Forward primer 2 (5’ to 3’): GTTCGTACCCCTCTCGAGAATA

Reverse primer 2 (5’ to 3’): CCATCCACTTCTCATCTGAAAGACC

The PCR products were purified by using QIAquick PCR Purification Kit (QIAGEN, #28104). The resulting DNA samples (∼20 ng/μl) were submitted to GENEWIZ for deep sequencing through the Amplicon-EZ sequencing pipeline (without lllumina^®^ partial adapters).

To analyze the deep sequencing data, reads were first trimmed (Trimmomatic-0.39). Next, reads with a lc-dust scores lower than 0.07 were removed (PRINSEQ++, version 1.2). Only reads containing an unchanged sequence of either primer used for the final amplicon (Forward and Reverse primers 2) were next selected and trimmed to their 5’ ends. All reads shorter than 220 bp and reads supporting no-deletion events were discarded. Remaining reads were sorted and grouped by common sequences. Junction positions of most common deletion events were manually verified and used for alignment (tolerance of two errors) of remaining reads. The custom code used for the analysis is available through GitHub (https://github.com/malkovalab/DeepSeqTools).

### Determining the frequency of IR-mediated deletions by droplet digital PCR (ddPCR)

Yeast cells containing the Ori1 or Ori2 *lys2-InsH* reporters at the 16 kb position were used to perform BIR-induction experiments as described in (74,76). 1.5 ml of yeast cultures with 5 × 10^7^cells/ml were collected by centrifugation 12 h post-BIR induction.

The cells were resuspended in 1 ml Spheroplasting buffer (0.4 M sorbitol, 0.4 M KCl, 40 mM sodium phosphate buffer pH 7.2, 0.5 mM MgCl_2_), then digested by addition of 5 μl Zymolyase buffer (0.1 g/ml 20 T zymolyase (MP Biomedicals, #08320921) dissolved in 2% glucose, 50 mM Tris–HCl, pH 7.5), and incubated at 37°C for 15 min. After that, cells were collected by centrifugation at 3000 rpm for 2 min with all liquid removed. Cells were then resuspended by addition of 500 μl 1× Cut smart buffer (50 mM potassium acetate, 20 mM Tris-acetate, 10 mM magnesium acetate, 100 μg/ml BSA, pH 7.9 at 25°C). Next, 5 μl 10% SDS buffer and glass beads (about 300 μl volume) were added to the resuspended cells and then suspensions were vortexed for 1 min to break all cells. To remove RNA, 20 μl 10 mg/ml RNase buffer was added to the mixture, which was then incubated at 37°C for 30 min. After incubation, 500 μl phenol:chloroform:isoamyl alcohol (25:24:1) (Fisher, #15593-049) were added to the mixture followed by brief vortexing and centrifugation at 13 000rpm for 15 min. The upper layer was transferred to a new tube by careful pipetting. Another round of wash by phenol:chloroform:isoamyl alcohol (25:24:1) followed by centrifugation was repeated. The upper layer after centrifugation was transferred to a new tube and mixed with 50 μl 3M sodium acetate buffer (PH 5.2) and 500 μl isopropanol. After brief vortexing, the mixture was kept at room temperature for 20 min and then centrifuged at 13 000 rpm for 20 min. All liquids were removed without touching the pellet of DNA. Then 500 μl 80% ethanol were added to the tube followed by centrifugation at 13 000 rpm for 5 min. All liquid was removed, and pellets were left to air dry for at least 10 min. 50 μl water was added to dissolve the dried DNA, which was then quantified by Qubit and stored at –20°C if not immediately used.

To detect Type I events, 2 μl of undiluted DNA was mixed with 10 μl 2× ddPCR supermix for probe (no dUTP Biorad 1863023), 7 μl water, and 1 μl of 20× primer sets that specifically recognize the junction formed in Type I events (see sequences below). To determine the amount of yeast genomic DNA, the original DNA solutions were diluted by serial dilution to ∼0.2 ng/μl, and then 2 μl of the diluted DNA were mixed with 10μl 2× ddPCR supermix for probe (no dUTP), 7μl water, and 1 μl of 20× *ACT1* primer set (sequences are listed below). The PCR mixture and Droplet generation oil (Biorad, #1863005) were used to generate droplets using QX200 Droplet Digital PCR (ddPCR) System. PCR reactions for both Type I events and for the *ACT1* locus were conducted with the following program: step 1: 95°C for 10 min; step 2: 94°C for 30 s (2°C change per seconds), 60°C for 2min 30 s (2°C change per second); step 3, repeat step 2 for 39 cycles; step 4: 98°C for 10 min; step 5: 12°C for 30 min. After PCR, the reactions were analyzed by QX200 Droplet Digital PCR (ddPCR) System. The Type I events were analyzed by channel 1 to detect the FAM signal, while the *ACT1* locus was analyzed by channel 2 to detect the HEX signal. The sequences for the primers sets (ordered from IDT) and used for the ddPCR analysis were as follows:

*Type1* (ratio of primers and probe was 4:1 and the probe was labeled by FAM):

Forward (5’ to 3’): CTGTGTTTGCCAAATCCATCC

Reverse (5’ to 3’): TCTTATACACAAGTAGCGTCAG

Probe (5’ to 3’): AGGCAGGTATCACCTATGGTACTTGGA.

*ACT1* (ratio of primers and probe was 4:1 and the probe was labeled by HEX):

Forward (5’ to 3’): GCCTTCTACGTTTCCATCCA

Reverse (5’ to 3’): GGTAGAGAGAAACCAGCGTAAA

Probe (5’ to 3’): TTCCGGTGATGGTGTTACTCACGT

## RESULTS

### BIR promotes deletions between microhomologies flanking inverted DNA repeats

**Figure 1.**
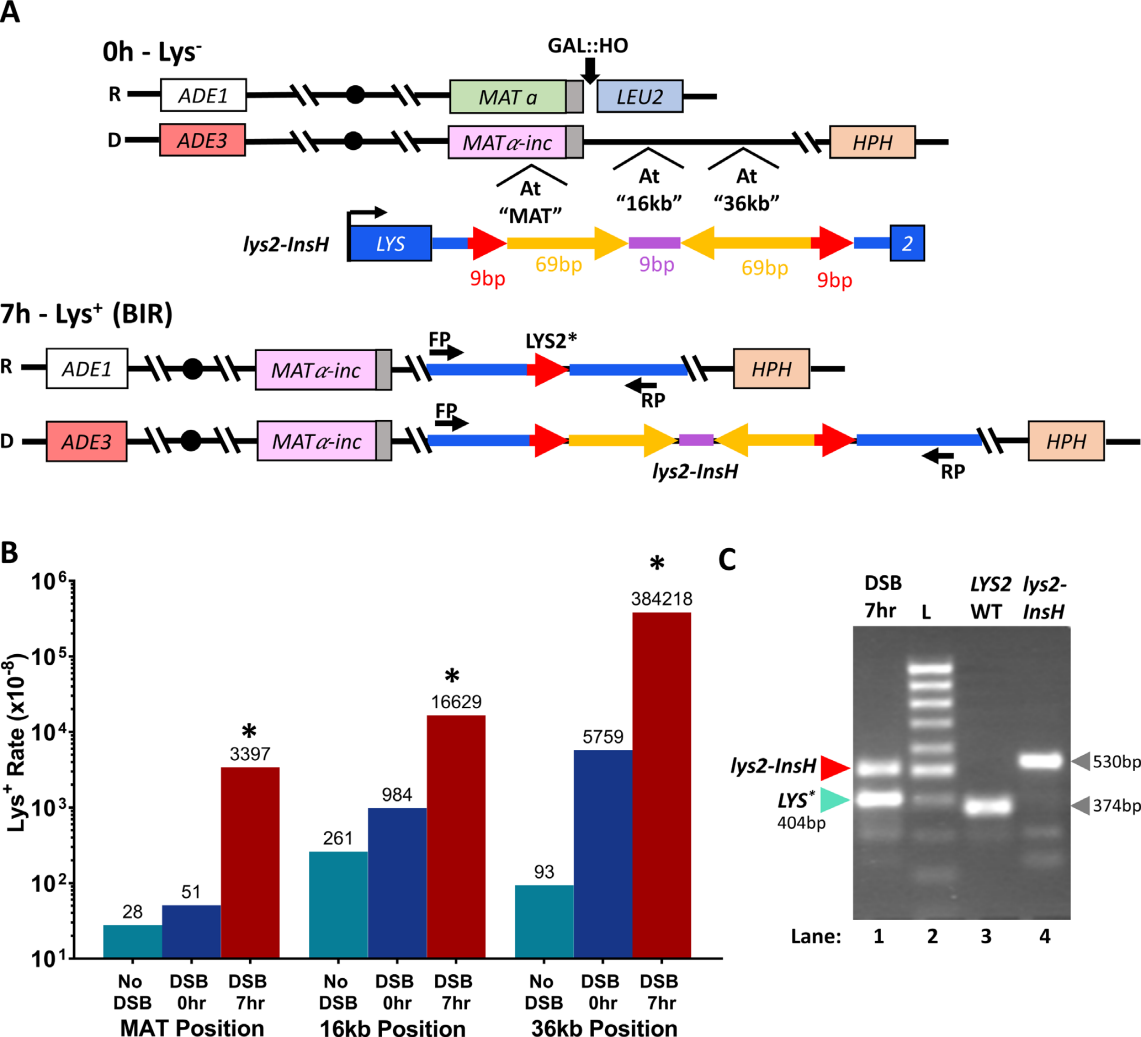
BIR promotes deletions between microhomologies flanking inverted DNA repeats. (**A**) The *lys2-InsH* reporter before (0 h) and after (7 h) DSB. **Top**. BIR is initiated by induction of a DSB by HO endonuclease at *MATa* located in a truncated (recipient) chromosome (R) of yeast disomic for Chr. III. Invasion of one broken end of the recipient into the homologous full-length donor (D) chromosome containing *MATα-inc* establishes BIR synthesis that can progress approximately 100kb to the end of the chromosome. The *lys2-InsH* (Lys^−^) reporter consists of 69bp inverted repeats (yellow) separated by a 9bp spacer (purple) and is flanked by 9bp direct repeats (red). The construct was inserted at three different positions along the track of BIR synthesis in the donor chromosome: “*MAT*” (at *MATα-inc*), “16kb” centromere distal from *MATα-inc*, and “36 kb” centromere distal from *MATα-inc*. **Bottom**. Replication slippage during BIR proceeding through the *lys2-InsH* reporter can result in deletion of InsH producing a functional allele (LYS2*) conferring a Lys^+^ phenotype. Positions of primers (FP: forward primer, RP: reverse primer) for confirming deletions by PCR are shown for both LYS2* and *lys2-InsH* alleles. (**B**) Lys^+^ reversion rates for the strains containing *lys2-InsH* reporter cassette at the three positions specified in (A) prior to DSB induction (DSB 0hr), following BIR (DSB 7 h), as well as in isogenic strains lacking the HO cut-site (No DSB). Asterisks indicate statistically significant difference (*P* < 0.005) from Lys^+^ reversion rates at 0 h (DSB 0 h). Numbers above bars indicate median rates (see Supplementary Data S2 for precise *P*-values and 95% CIs). (**C**) Representative gel image showing PCR-amplified products of the *lys2-InsH* allele and the LYS2* allele (404 bp) present in a single BIR outcome (lane 1). PCR products of WT *LYS2* (374 bp) (lane 3) and the *lys2-InsH* construct before BIR (530 bp) (lane 4) are also shown for comparison, along with a 100-bp DNA ladder (L) in lane 2. Primers used for all PCR products are those shown in (A, bottom) (FP and RP). Analysis of *lys2-InsH* deletion products (like LYS2*) was performed by Sanger sequencing.

When IRs are included into single-stranded DNA (ssDNA) they form secondary DNA structures (hairpins) promoting replication stalling and polymerase slippage at microhomologies leading to deletions between them. It is believed that the frequency of replication slippage-induced deletions is determined by the frequency of hairpin formation, and therefore by the persistence of ssDNA (26,34–36). Because BIR is a source of long, persistent ssDNA (4,21,51), we asked whether this ssDNA would promote hairpin-induced deletions at microhomologies flanking IRs placed on the synthesis track of BIR. To accomplish this, we used our BIR experimental system in *Saccharomyces cerevisiae* disomic for chromosome III (64). In this system, a galactose-inducible HO-endonuclease initiates a DSB at the *MATa* locus on a Chromosome III (Chr III) that is truncated by the insertion of *LEU2* and telomere sequence centromere distal to *MAT*. BIR is the dominant repair pathway in this system and is initiated by 5’ to 3’ resection that can proceed for long distances (up to the centromere) followed by invasion of the broken chromosome into the homologous region of the full copy of Chr III (the donor) that contains a *MATα-inc* allele that cannot be cut by HO-endonuclease (Figure 1A). Because the length of resection is variable, the exact site of invasion is not known, but it most often occurs within 3 kb centromere proximal to the Y region of the *MAT* locus of the donor chromosome (64). Strand invasion is followed by removal of a flap structure formed by the 3' tail that includes at least 650 bp of sequence that is non-homologous to the donor chromosome. This is then followed by the beginning of DNA synthesis. The major outcome of DSB repair in this system is two full copies of Chr III, where the newly synthesized DNA is conservatively inherited (51, 62). To assay whether the propensity for hairpin-induced deletions in ssDNA formed by BIR exceeds the level previously observed in S-phase, we placed a *lys2-InsH* reversion reporter (*InsH* is an IR-containing insertion within the *LYS2* gene) (36) on the track of BIR synthesis at three positions (Figure 1A). InsH was previously shown to trigger deletions resulting from replication slippage at microhomologies flanking IRs (34,36) and intrachromosomal recombination (35) during S-phase in yeast. The InsH sequence consists of two 69 bp inverted repeats separated by a 9 bp spacer and is flanked on both sides by two 9 bp direct repeats originating from the *LYS2* sequence (Figure 1A). InsH is inserted in the terminal region of the *LYS2* gene (similar to (36)), and the resulting *lys2-InsH* reporter construct yields a non-functional alpha aminoadipate reductase protein, resultng in a Lys^−^ phenotype. In-frame deleton of InsH from the *lys2-InsH* reporter can yield a functional *LYS2* gene (36), thereby allowing us to assess the deletion frequency (Figure 1A).

We hypothesized that InsH could readily form a hairpin in the long ssDNA that accumulates behind the BIR migrating bubble (D-loop) during leading strand synthesis, which is known to be a major source of mutagenesis during BIR (4,21,51,61,62). If lagging strand BIR synthesis encounters a stable hairpin in the nascent template strand, we predicted a high level of Lys^+^ reversion following BIR slippage between the 9 bp direct repeats flanking the IRs following the hairpin formation in the ssDNA serving as a template for lagging strand BIR synthesis. To test this, we induced BIR by addition of galactose to liquid cultures of yeast that carried the *lys2-InsH* reporter at *MAT* (see Materials and Methods for details), at 16 kb, or at 36 kb from the HO-site. At all positions, the *lys2-InsH* reporter was oriented such that transcription was co-directional with the direction of BIR synthesis (Figure 1A). The frequency of Lys^+^ reversions was measured before BIR (by plating yeast cultures on Sc-Lys drop-out media prior to galactose addition) as well as after BIR (by plating 7 h after DSB induction with galactose). We observed that the rate of Lys^+^ reversions (resulting from InsH deletions) at all three positions was significantly (17-67x) higher after BIR (Figure 1B, DSB 7 h) than the rate of Lys^+^ reversions prior to galactose addition (Figure 1B, DSB 0 h), and 64-4131x higher than controls lacking the HO-cut site (No DSB) and without galactose added (similar to (65), see Materials and Methods for details) (Figure 1B, no DSB, Supplementary Data S2). We next assessed the size of the deletions that produced Lys^+^ reversions in 7hr DSB outcomes for the 16 kb position reporter by PCR. We observed (Figure 1C) that Lys^+^ outcomes typically contained two bands (one corresponding to the donor copy of *lys2-InsH* that remained unchanged (530 bp band) and a second, deletion product, that was shorter than *lys2-InsH*). Two distinct classes of the deletion products were observed by PCR: those that matched the expected size of the *LYS2* fragment following the full deletion of the InsH sequence (374 bp band), and those where the deletion was slightly less than the full InsH sequence length, creating a longer PCR product than that observed for the full InsH deletion (Figure 1C, LYS2*).

### The polarity of insH deletions induced by BIR

**Figure 2.**
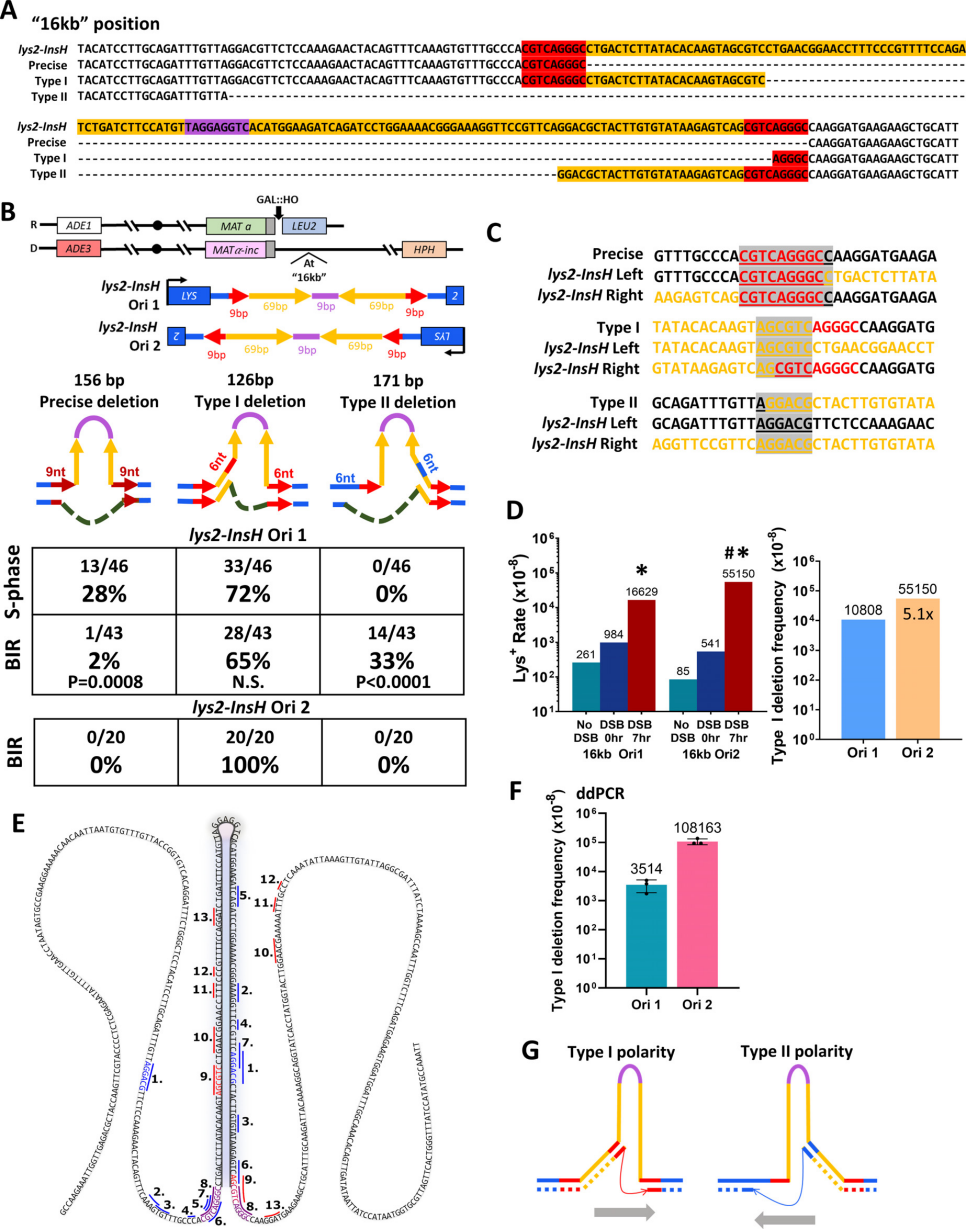
(**A**) Sequences of full *lys2-InsH* reporter and its derivatives following precise (restoring the wild type *LYS2* allele) and imprecise (Type I and Type II) deletions of InsH. Colors correspond to those depicted in Figure 1A from the full-length *lys2-InsH*. (**B**) Distribution of various types of InsH deletions among Lys^+^ revertants obtained in strains with the *lys2-InsH* reporter at 16kb in Orientation 1 (Ori1) or Orientation 2 (Ori2). (Upper) Schematic of *lys2-InsH* reporter inserted at 16kb in Orientation1 (Ori1) and Orientation 2 (Ori2). (Lower) Schematics of deletion types in Lys^+^ outcomes with respect to the hairpin structure formed by the InsH insertion in *LYS2* at 16kb in Ori1 and Ori2. These deletion types generate an in-frame *LYS2* gene (126, 156 and 171 bp deletions are all divisible by 3). “S-phase”: deletions that occurred in No DSB strains. “BIR”: deletions among Lys^+^ revertants obtained after BIR. *P*-values are shown to indicate statistically significant differences (*P* < 0.05) measured by Fisher's exact test for the deletion type fraction during BIR compared to S-phase. N.S. = no significant difference (*P* ≥ 0.05). (**C**) Alignment of *lys2-InsH* deletion breakpoints for precise, Type I, and Type II deletions to left and right sides of the full *lys2-InsH* sequence. Colors correspond to those depicted in A from the full length *lys2-InsH* allele in the donor chromosome. Gray highlighted and underlined bases are indicative of breakpoint microhomologies. (**D**) Left. Lys^+^ reversion frequencies of the Ori1 and Ori2 reporters. The data for Ori1 reporter are the same as shown in Figure 1B. Asterisks indicate statistically significant difference (*P* < 0.0001) from Lys^+^ reversion rates at 0 h (DSB 0 h). Pound symbol indicates statistically significant difference (*P* < 0.0001) from Lys^+^ reversion rate in Ori1 at 7 h (DSB 7 h). Right. Frequencies of Type I deletions after BIR in Ori1 and Ori2 reporter strains. Frequencies (shown above bars) were calculated by multiplying the fraction of BIR-associated Type I mutations (in B) by the rates shown in (D). Fold increase of Ori2 Type I over Ori1 Type I is shown inside the bar for Ori2. (**E**) Summary of InsH deletion types identified by deep sequencing. The numbers correspond to the types in Supplementary Data S3 and S4. The colored lines indicate microhomologies at deletion breakpoints: red – Type I-like, blue – Type II-like. Colored sequence indicates microhomologies at breakpoints: red – Type I deletion, blue – Type II deletion, purple – precise deletion. (**F**) Frequencies of Type I deletions in the *lys2-InsH* Ori1 and Ori2 reporters as determined by ddPCR experiments. Averages ± SD based on the results of three experiments are shown. (**G**) Schematic of InsH deletion polarities for observed deletion types. Gray arrows indicate the implied direction of synthesis. The colors of microhomologies are similar to (E).

Previous studies reported the precise excision (deletion) of InsH that occurred during S-phase DNA replication and was mediated by replication slippage at the flanking 9 bp direct repeats of the *LYS2* sequence, yielding a wild-type *LYS2* allele (34,36). Because we observed that PCR products from 7 h Lys^+^ revertants often did not match the wild-type *LYS2* PCR product by size, we asked whether deletions of InsH formed during BIR synthesis are imprecise. We Sanger sequenced 7hr Lys^+^ BIR outcomes from the 16 kb *lys2-InsH* reporter strain and Lys^+^ isolates from isogenic “no DSB” strains for comparison. We observed that the majority of InsH deletions during BIR were imprecise and asymmetrical such that the deletion products retained a part of the InsH quasi-palindrome; the remaining sequence was either from the left inverted repeat (Type I deletion) or from the right inverted repeat (Type II deletion) (Figure 2A–C). The Type II deletion also resulted in a loss of a portion of the *LYS2* gene sequence, yet still produced a Lys^+^ phenotype, despite slower growth of colonies on synthetic media lacking Lysine (Figure 2A, B, C). All three of the deletion types observed (Precise, Type I and Type II) produced an in-frame *LYS2* gene sequence (Figure 2A, B) and contained microhomologies on their breakpoints (9-bp-long for precise deletions and 6-bp-long for imprecise deletions) (Figure 2C). Of the Lys^+^ isolates from the “No DSB” strains (reflecting deletions resulting from S-phase DNA replication), there were significantly more precise deletions of InsH as compared to BIR (Figure 2B), though the majority were still imprecise Type I deletions. Importantly, all three deletion types (precise, Type I and Type II) were stimulated by BIR, while Type I occurred most frequently. When we inserted *lys2-InsH* at the same position (16 kb) in inverted orientation (Ori2) with respect to that of the original strain (Ori1) (see schematic in Figure 2B), the frequency of BIR-associated Lys^+^ reversions was increased 3.3 times as compared to what we observed in Ori1 (Figure 2D), and all of the Lys^+^ outcomes (20/20) analyzed by Sanger sequencing) were Type I (Figure 2B). By direct comparison, Type I frequencies were 5.1× more frequent during BIR with the Ori2 reporter than with the Ori1 reporter, even though in both orientations Type I deletions were greatly stimulated by BIR (Figure 2D). We believe that the lack of Type II events among Ori2 outcomes results from a significant decrease in the fraction of Type II events (from 33% among Ori1 outcomes to ∼2% among Ori2 outcomes). This calculation is based on the 5.1× increase in Type I events (from leading to lagging strand (Figure 2D)) and therefore on 5.1× decrease of Type II events (from lagging to leading strand).

We also performed Sanger sequencing of Lys^+^ BIR outcomes from Ori1 *MAT* and 36 kb reporter positions and found that they produced only Type I imprecise deletions (Supplementary Figure 1A). To assess whether the ability for the reporter to produce a Lys^+^ phenotype may differ between reporter positions (and could result, for example from differences in the levels of *LYS2* expression between different reporter locations), we transformed strains with reporters in different positions with both Type I and Type II imprecise deletion fragments obtained from the 16kb Ori1 strain. Indeed, we found that only Type I deletion fragments supported a Lys^+^ phenotype at *MAT* and 36 kb positions (even though with varying efficiency), while both Type I and Type II fragments did so at 16 kb in the Ori1 strain (Supplementary Figure 1B). Due to the possibility that the selection for Lys^+^ reversions could be affected by the level of *LYS2* expression for two orientations of the reporter as well, and also because Lys^+^ selection precludes identification of any deletions that inherently do not produce Lys^+^ outcomes (e.g. out of frame deletions), we next analyzed non-selected post-BIR cells by deep sequencing. Specifically, yeast cultures were collected 12 h following DSB induction, at the point when BIR was expected to complete in the majority of the cells. The DNA purified from these cells was subjected to PCR amplification and deep sequencing of the *lys2-InsH* region followed by identification of reads containing deletions of InsH. This method allowed us to reveal the main types of deletions missed in reporter experiments using Lys^+^ selection and to identify the most frequent deletion types among them.

By deep sequencing, we detected Type I (which was the most frequent following BIR in both Ori1 and Ori2 reporters), Type II, and precise deletion types, and we detected several new types not seen in reporter experiments (Figure 2E, Supplementary Data S3, S4). One of the more frequent new types that we detected, which we called J1 (type 7 in Figure 2E; Supplementary Data S3, S4), resulted from deletion of 125 nucleotides and contained 5bp-microhomologies on its boundaries (Figure 2E; Supplementary Data S3, S4). Because this deletion was not in-frame it could not produce a Lys^+^ outcome but appeared to be a major type due to its relative frequency among the other deletion classes.

One limitation of the deep sequencing method is that it cannot yield accurate absolute frequencies of BIR-induced deletion events due to the requirement for standard PCR amplification, which can introduce bias among deletion outcomes and is biased against outcomes without deletions that contain the hairpin-forming IR and thus do not amplify faithfully to calculate their frequency in the cell population. To address this limitation, we next used a highly sensitive digital droplet PCR (ddPCR) method to detect the absolute frequencies of the most abundant deletion types during BIR. Although the frequencies of Type II and J1 deletions were below the threshold for ddPCR detection, the Type I deletion was detectable, and we determined that the frequencies of Type I during BIR were ∼4 × 10^−5^ in Ori1 and ∼1 × 10^−3^ in Ori2 (Figure 2F, Supplementary Figure 2). Importantly, the frequencies of Type I for No-DSB controls for both Ori1 and Ori2 were below ddPCR detection threshold (Supplementary Figure 2), thus confirming that the Type I frequencies calculated by ddPCR following BIR were indeed BIR-specific.

Together, we conclude that BIR promotes deletions at microhomologies flanking IRs. The majority of these deletions have a polarity (with one microhomology located inside the inverted repeat and another outside of the repeat (Figure 2E, G). This type of polarity was previously observed for similar events mediated by Polδ during S-phase lagging strand synthesis (36). There it was proposed that following hairpin formation, Polδ can copy via displacement synthesis inside a hairpin but undergoes frequent template switches from inside to the outside the hairpin (36). It was also proposed that the polarity of the deletion events reflects the direction of synthesis (from the microhomology inside the hairpin towards one outside). Using the same logic for BIR, we propose that Type I events result from slippage during lagging strand synthesis in Ori2 and during leading strand synthesis in Ori1 (Figure 2G, see also Discussion and the Figure 6B for details). Even though the frequency of the latter is lower than the frequency of the former, our data suggest that ssDNA in the template for leading strand synthesis forms and persists, which was not previously appreciated.

### The effect of polymerase mutations on InsH deletions

**Figure 3.**
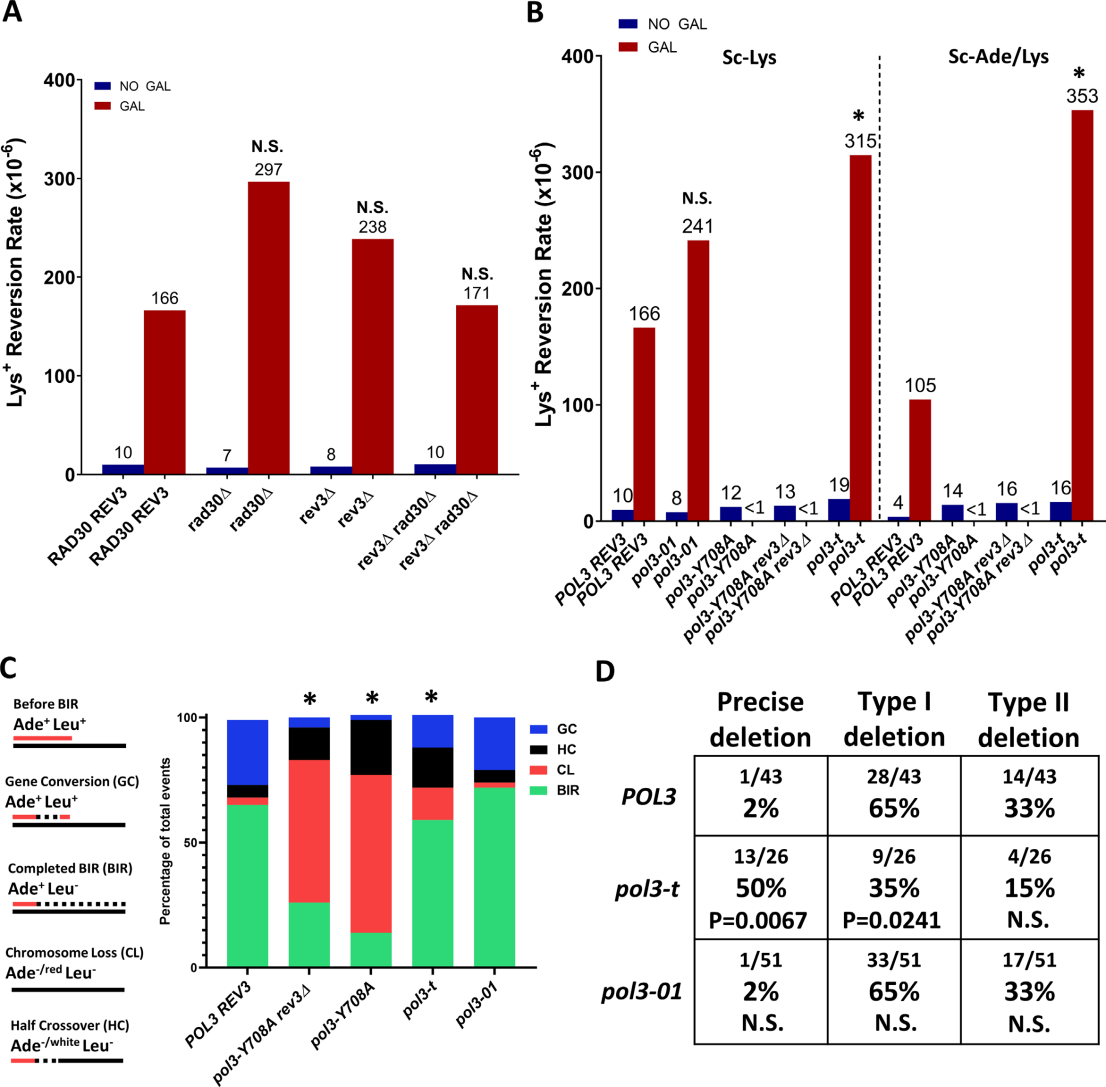
The effect of polymerase mutations on InsH deletions. (**A**) Eliminating Pol η (*rad30*Δ) or Pol ζ (*rev3*Δ) does not affect the frequency of Lys^+^ resulting from deletions in *lys2-InsH* at 16kb during BIR. “NO GAL” = rates prior to the addition of galactose. “GAL” = Lys^+^ rates following BIR. Median values are listed above the bars. N.S. = not significantly different from wild-type (*RAD30 REV3)* (P≥0.05 determined by Mann-Whitney U test, see Supplementary Data S5 for P-values and 95% CI). (**B**) The effects of mutations in *POL3*, including: *pol3-01*, *pol3-Y708A*, and *pol3-t* on Lys^+^ reversion rate after BIR induction in strains harboring the *lys2-InsH* reporter at the 16kb position. Sc-Lys: Lys^+^ were selected for lysine prototrophy only. Sc-Ade/Lys: Lys^+^ were selected for lysine and adenine prototrophy to eliminate chromosome loss and half-crossover outcomes which exhibit adenine auxotrophy. Asterisks indicate values that were significantly different (*P* < 0.01) from wild-type (*POL3*) and N.S. indicates no significant difference (*P* ≥ 0.05) from wild-type (*POL3*). “<1” indicates that rates were not calculable (see Supplementary Data S5 for frequencies used in rate calculation). Other details similar to (A). (**C**) BIR efficiency is reduced (and chromosome loss and half-crossovers increased) in *pol3-Y708A* and *pol3-t* mutants as compared to *POL3*. BIR efficiency is not affected in *pol3-01*. Asterisks indicate significant differences in DSB repair outcome fractions from *POL3* (*P* < 0.05) measured by Fisher's exact test. Different DSB repair outcomes are illustrated in the schematic (left). (**D**) *lys2-InsH* deletion spectra in *POL3*, *pol3-t*, and *pol3-01* strains. *P*-values are listed to indicate statistically significant differences (*P* < 0.05) in the fractions of individual deletion types compared to *POL3* measured by Fisher's exact test. N.S. = no significant difference (*P* ≥ 0.05).

Pol δ was recently confirmed as the main replicative polymerase driving both leading and lagging strand synthesis during BIR (77), which makes it likely that it also mediates deletions of InsH. Yet, stalling of Pol δ during BIR was proposed to recruit translesion polymerase ζ (Pol ζ) to mediate template switching at microhomology (75). Because the deletions of InsH that we observed during BIR were mediated by microhomology, we sought to determine whether a similar switch from Pol δ to Pol ζ might also take place in the process of InsH deletion. We observed that deletion of *REV3*, encoding the catalytic subunit of Pol ζ, had no significant effect on the rate of Lys^+^ reversions of the *lys2-InsH* reporter at the 16 kb position during BIR (Figure 3A, Supplementary Data S2). To assess possible redundancy with Polymerase η (Pol η), another translesion polymerase that has the capacity to substitute for Pol δ at lesions (reviewed in (78)), we also created *rad30Δ* (deletion of the gene encoding the catalytic subunit of Pol η) and *rev3Δ rad30Δ* mutants. Neither of these mutations had any significant effect on the rate of Lys^+^ reversion of the *lys2-InsH* reporter after BIR as compared to the wild-type (Figure 3A, Supplementary Data S2). In addition, we did not observe any effect following the deletion of *POL4* (Supplementary Data S2).

Because we did not find any evidence supporting participation of translesion polymerases in the deletions of InsH, we next tested the effects of various mutations affecting Pol δ, the main polymerase driving both leading and lagging strand BIR synthesis (77). Three mutations *in POL3*, *pol3-t*, *pol3-Y708A* and *pol3-01* were selected for this analysis. In particular, *pol3-t* (Pol δ active site mutation (36,37,79)), was previously shown to greatly increase deletions at microhomologies promoted by inverted repeats in S-phase (36,37). A second mutation, *pol3-Y708A* (Pol δ nucleotide binding pocket mutation (71)), was characterized as having a mutator phenotype dependent on Pol ζ (71,80,81). This mutation was shown to decrease processivity during BIR, leading to increased half-crossover outcomes with reduced BIR efficiency (82), but the distance that Pol δ carrying the *pol3-Y708A* mutation could synthesize during BIR remained unknown. A third mutation, *pol3-01*, leads to proofreading deficiency of Pol δ (83) and was shown to eliminate frameshifts and complex events involving template switches during gene conversion (84). Based on these data and on preceding investigations (85), the *pol3-01* mutant was hypothesized to create a more processive Pol δ during BIR. Therefore, we hypothesized that if this is true, then a more processive Pol δ might displace secondary structures more easily, and therefore affect the frequency of deletions and their spectrum in our system.

For *pol3-Y708A* (and for *pol3-Y708A rev3Δ*) mutants, we observed that BIR synthesis never reaches the InsH position (16kb) in our system. This was concluded based on a very high frequency of CL and HC (defective DSB repair outcomes) (Figure 3C), which was consistent with previous observations (82), as well as on a very low level of Lys^+^ reversions which did not increase following DSB induction (Figure 3B, Supplementary Data S5). Based on these observations, we concluded that even Ade^+^ Leu^−^ colonies that were observed in this mutant were not completed BIR events, but rather aberrant repair outcomes (similar to those described in (82)), where BIR synthesis was interrupted before reaching the 16kb position.

Next, Lys^+^ reversion rate in the *pol3-01* mutant was not significantly different from the wild-type *POL3* strain (Figure 3B), nor did this mutation have any effect on BIR efficiency (Figure 3C). Additionally, the *pol3-01* mutant showed no difference in InsH deletion spectrum as compared to the spectrum of the wild-type *POL3* strain (Figure 3D). Combined with Lys^+^ reversion frequency data for this mutant, our results do not show any evidence of increased displacement activity of Pol δ in the *pol3-01* mutant in our BIR system.

Finally, we observed that in *pol3-t* mutants, Lys^+^ reversion rate during BIR was modestly (1.9×), but significantly increased as compared to the wild-type *POL3* strain, indicating a higher rate of InsH deletion (Figure 3B, left (Sc-Lys)). As expected, BIR efficiency was also reduced in *pol3-t* mutants and the frequency of chromosome loss (CL) and half-crossover (HC) outcomes was increased, (Figure 3C), consistent with previous observations (82). When experiments were carried out in the absence of adenine to eliminate CL and HC outcomes (see Figure 3C, schematic), *pol3-t* Lys^+^ reversion rate was more dramatically increased (3.4x) as compared to wild-type *POL3* strains (Figure 3B, right (Sc-Ade/Lys)). In addition, we observed that *pol3-t* mutation altered the distribution of deletions, such that precise deletions were favored, which was different from the spectrum observed in wild-type *POL3* where Type I imprecise deletions were predominant (Figure 3D). One possible explanation for this change was that progression of synthesis by *pol3-t* is kinetically slower than *POL3* and this allows the formation of longer ssDNA regions allowing the full InsH hairpin to form more often. However, when BIR was induced in *POL3* (wt) strains at 20°C (which could also slow down BIR progression), Lys^+^ reversion rate and the spectrum of InsH deletions were similar to what we observed at 30°C and did not tend towards the rate and spectrum observed in the *pol3-t* mutant (Supplementary Figure 3, Supplementary Data S2).

### ssDNA in the template for BIR leading strand synthesis is susceptible to APOBEC3A deamination

**Figure 4.**
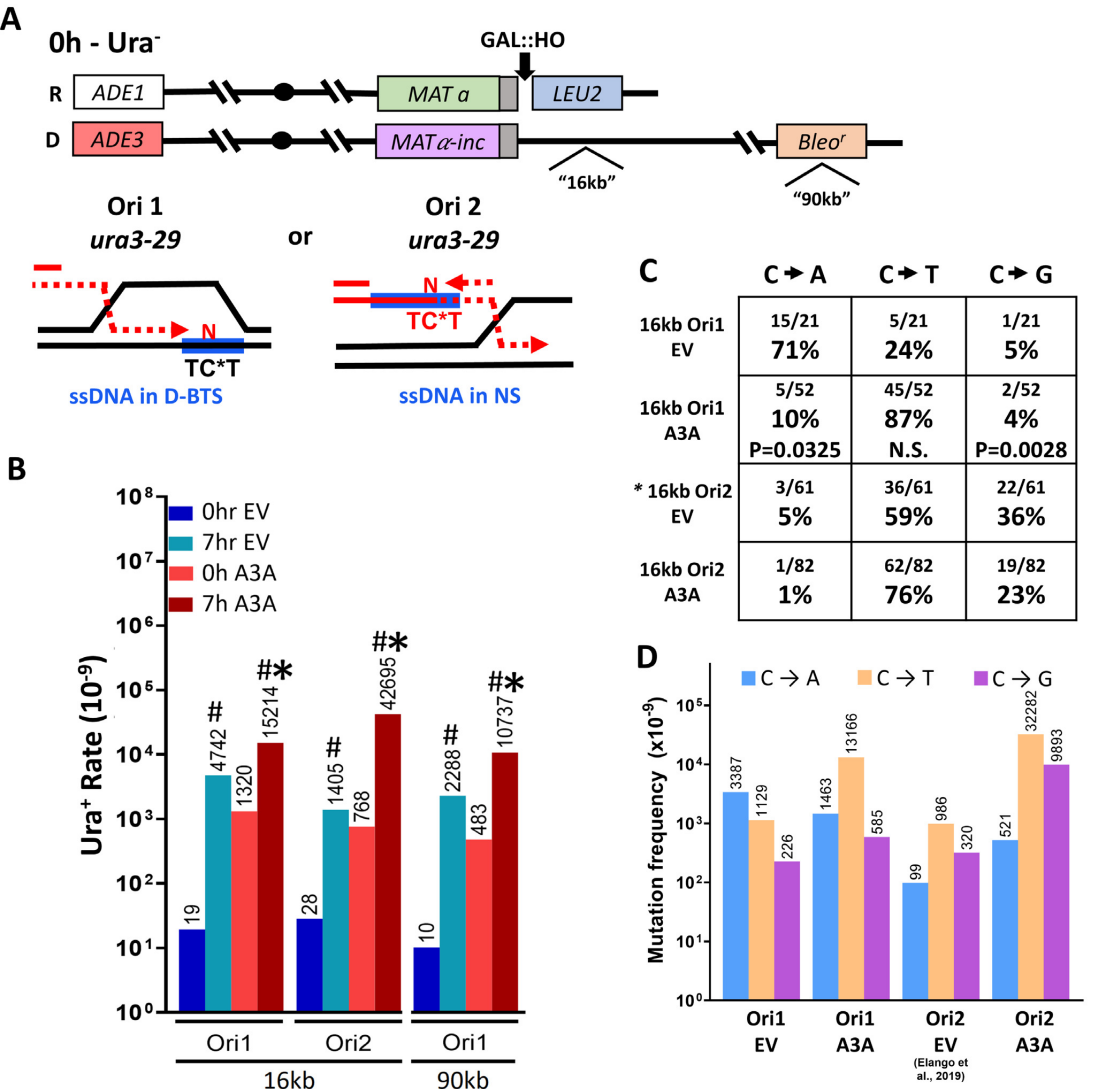
ssDNA in the D-BTS is susceptible to mutagenesis induced by APOBEC3A. (**A**) **Top**. Yeast disomic BIR system similar to that shown in Figure 1A, but with the *ura3-29* (base substitution) reporter inserted at the 16kb and 90kb positions in two orientations (Ori1 and Ori2). Note: strains with *ura3-29* at 16kb contained *KanMX* at 90 kb position instead of Bleo^r^. Prior to BIR (0h), the strain is Ura^−^. **Bottom**. schematic showing location of TCT motif recognized by A3A in the template for the BIR leading strand ssDNA of the Ori1 *ura3-29* reporter (ssDNA in D-BTS), and in the ssDNA in the template for the lagging strand of the Ori2 *ura3-29* reporter (ssDNA in nascent strand (NS)). Blue rectangles indicate ssDNA. Cytidine deamination (indicated with an asterisk) in either of these locations can produce reversion to a Ura^+^ phenotype if any base other than G is incorporated. (**B**) Rates (solid bars) of Ura^+^ reversions before (0 h) and after BIR (7 h), in the presence of a plasmid containing APOBEC3A (A3A) or empty vector (EV) in *UNG1* strains with *ura3-29* reporter in Ori1 and Ori2 at 16 kb and Ori1 at 90 kb. Median values are listed above each bar. Significant differences (*P* < 0.005) for the comparisons of A3A and EV strains following BIR (7 h) are marked with asterisks. Pound symbols indicate significant differences (*P* < 0.01) for the comparison of A3A- or EV-containing strains to their respective pre-BIR (0 h) levels. See Supplementary Data S6 for *P*-values, 95% CIs and details on rate calculation. (**C**) Ura^+^ mutation spectra in Ori1 and Ori2 reporter strains expressing A3A or EV during BIR in the *UNG1* (wild type) background. Asterisk indicates data from (21). *P*-values are listed to indicate statistically significant differences (*P* < 0.05) of Ori1 A3A from Ori2 A3A spectra. N.S. = no significant difference. (**D**) Frequencies of individual substitution mutations after BIR in the *UNG1* (wild type) strain with Ori1 or Ori2 reporters. Ori2 EV spectra data used are from (21). Frequencies were calculated by multiplying the fraction of each mutation type (in C) by the rates shown in B, and statistics are shown in (B) and (C).

The high frequency of Type I deletions in Ori1 orientation of insH during BIR, allowed us to hypothesize that ample ssDNA is exposed in the template for BIR within the D-loop structure. Therefore, we asked whether this ssDNA can be detected by its susceptibility to APOBEC-induced damage. Previously, we expressed APOBEC3A (A3A), a cytosine deaminase that converts cytidine in the context of ssDNA into deoxyuridine (dU), in yeast cells undergoing BIR (21). This led to the formation of long mutation clusters on the track of BIR and allowed us to conclude that long stretches of ssDNA accumulate behind the BIR migrating bubble formed by leading strand BIR synthesis and by resection of the DSB end. However, in those studies we never asked whether mutagenic ssDNA can also accumulate within the D-loop bottom template strand (D-BTS). To investigate this, we placed a *ura3-29* base substitution reporter cassette (86) at the 16kb position centromere-distal from *MATα-inc* in the donor chromosome of our disomic galactose-inducible DSB system (Figure 4A). The *ura3-29* reporter contains a T to C transition at position 257 in the *URA3* gene, resulting in a Phe to Ser amino acid change that yields a Ura^−^ phenotype (86). This mutation can revert to a Ura^+^ phenotype by a C to T, C to G, or C to A base substitution (51,86). Importantly, the cytosine in the mutant position of *ura3-29* is located within a TCW motif recognized by A3A. We placed the *ura3-29* reporter in two orientations with respect to BIR progression. In the first orientation (Ori1), the TCW motif can become a target for A3A if ssDNA is formed in the D-BTS. The other orientation (Ori2) places the reporter cytosine in the nascent strand (NS) ssDNA that accumulates behind the BIR D-loop as a result of leading strand synthesis (Figure 4A, schematic). To express A3A in these reporter strains, we transformed them with a centromeric plasmid expressing A3A (22,63), and the same centromeric plasmid without A3A as an empty vector (EV) control. In the presence of A3A we observed a 3.2-fold increase in BIR-associated reversion of Ura^−^ to Ura^+^ for the Ori1 *ura3-29* reporter as compared to BIR-associated mutagenesis in the EV-harboring strains (Figure 4B, Supplementary Data S6). Because the reporter cytosine in Ori1 is most likely included into the ssDNA during BIR when it is formed in the D-BTS region (see Figure 4A, schematic), the observed increase implies that a significant amount of ssDNA is persisting during BIR in the D-BTS region. In strains harboring the *ura3-29* reporter in Ori2, we observed a 30-fold increase in Ura^+^ reversion rate during BIR in the presence of A3A as compared to EV (Figure 4B, Supplementary Data S6), consistent with the high mutagenicity of the long persistent NS ssDNA that we previously reported (21). To ensure that the A3A-induced increase seen in Ori1 strains after BIR was not unique to the 16 kb position of the reporter, we repeated the experiment with the Ori1 *ura3-29* reporter cassette at a position 90 kb centromere-distal from *MAT* (Figure 4A). We observed a similar 4.7-fold increase of Ura^+^ during BIR in the presence of A3A as compared to EV (Figure 4B, Supplementary Data S6).

### dU lesions in the D-BTS are poor substrates for error-free repair

Previously, we demonstrated that dU generated by A3A in the nascent ssDNA of BIR are frequently converted to AP sites by uracil-DNA glycosylase Ung1, and this conversion leads to reduction of mutagenesis via an error-free repair pathway (21). Here, we asked whether dU lesions generated in the D-BTS (see Figure 4A, schematic) are repaired through error-free pathways equally often. To this end, we tested the effect of deleting *UNG1* on A3A mutagenesis during BIR in strains harboring *ura3-29* reporter at 16-kb and 90-kb positions. Like what was previously observed for 90-kb position (21), the rate of Ura^+^ reversions increased dramatically (115-fold) in *ung1Δ* Ori2 strains as compared to wild-type (*UNG1* Ori2) for 16-kb position (Supplementary Data S6). However, such a dramatic increase was not observed in *ung1Δ* Ori1 strains (Supplementary Data S6). Rather, the 90-kb reporter position produced a rate of Ura^+^ that was only 4.2× higher in *ung1Δ* as compared to *UNG1*, which suggests that ∼24% of dUs introduced into the D-BTS led to mutations (as compared to only 5% of dUs that led to mutations when they were introduced into the ssDNA of the newly synthesized leading strand at the same location (Ori2 90 kb, see in (21)). The data obtained at the 16 kb position support this idea. Specifically, we did not observe an increase of Ura^+^ frequency following BIR in Ori1 *ung1Δ* strains as compared to the pre-BIR level, even though an increase was expected based on the observed increase in *ung1Δ* strains containing *ura3-29* in Ori2 (Supplementary Data S6). Also, when we analyzed the mutation spectra of BIR/A3A Ura^+^ revertants in *UNG1* strains with the reporter at this (16 kb) position, we observed significantly fewer C to G transversions (reflective of translesion synthesis across from abasic (AP) sites) than in Ori2 (Figure 4C). Further, our calculations (based on combining results shown in Figure 4B and C) demonstrated that, while the frequencies of both C to T and C to G substitutions were drastically increased following BIR in the presence of A3A (as compared to EV) in Ori2, only C to T substitutions were increased in Ori1 (BIR/A3A versus BIR/EV; Figure 4D). The lack of C to G increase in Ori1 suggests that dU lesions formed in the D-BTS region during BIR might be converted into AP sites more rarely by Ung1 as compared to lesions introduced into a nascent leading BIR strand (21).

**Figure 5.**
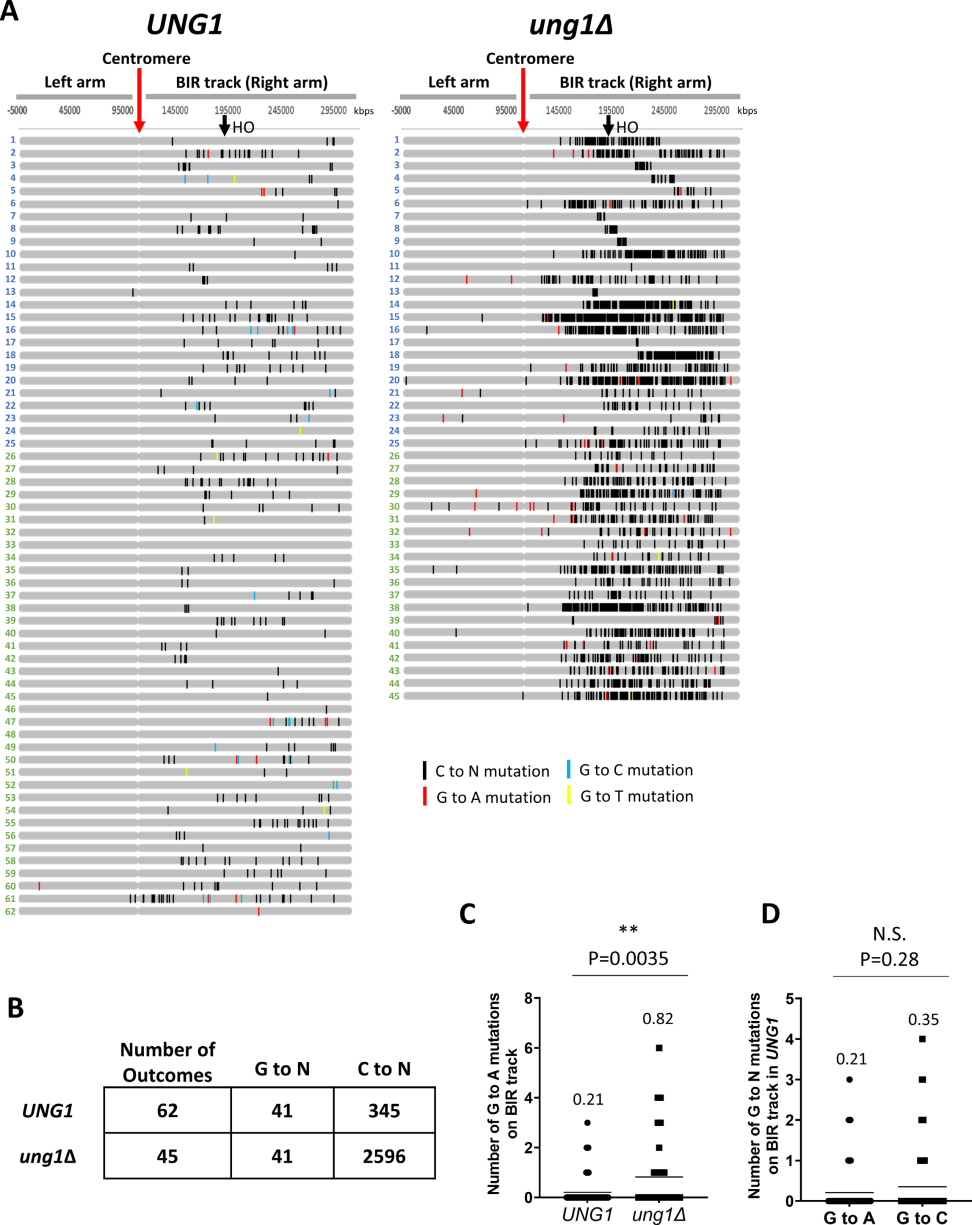
(**A**) A3A-induced mutations at cytosines (C to N: black tick-marks) and guanines (G to A: red, G to C: blue, and G to T: yellow tick-marks) on Chromosome III (Watson or 5’ to 3’ strand) identified by WGS of BIR outcomes from *UNG1* and *ung1Δ* backgrounds exposed to A3A. Outcomes numbered in blue or green are from independent experiments (see Supplementary Data S7 for details). (**B**) Summary of total number of G to N and C to N mutations on the right arm (BIR track) in BIR outcomes from *UNG1* and *ung1Δ* backgrounds shown in (A). (**C**) Number of G to A mutations per outcome on the track of BIR (right arm) in *UNG1* and *ung1Δ* backgrounds. Horizontal lines indicate mean values that are also listed above each plot. *P*-value obtained by unpaired *t*-test is listed above the plot to indicate statistical significance (asterisk) or no statistical significance (N.S.) of difference in the mutation number. (**D**) Number of G to N mutations per outcome (G to A or G to C) on the track of BIR (right arm) in the *UNG1* background. Mean values and statistical significance are as in (C).

We next asked whether A3A-induced damage in the D-BTS could be a significant contributor to mutagenesis along the entire track of BIR. To address this question, we performed whole genome sequencing (WGS) analysis for the outcomes of BIR exposed to A3A in both *UNG1* and *ung1Δ* strains. For these experiments, we combined previously analyzed BIR outcomes from (21) with outcomes from newly performed experiments (see Materials and Methods). We identified all base substitutions on Chr III and called C to N and G to N (with respect to the Watson strand) substitutions separately (see Supplementary Data S7). Because the exact site of invasion during BIR could occur anywhere along the length of resection (which can be up to the centromere (4,60,21)), we considered mutations called on the right arm of Chr. III to be part of the BIR repair track, while mutations called on the left arm were assumed to result from another source, such as S-phase replication (Figure 5A). On Chr III, C to N substitutions on the BIR track indicate mutations incorporated due to A3A damage to the nascent ssDNA that serves as a template for lagging strand synthesis. Meanwhile, G to N substitutions likely indicate mutations incorporated due to A3A damage to the D-BTS ssDNA. In total, based on sequencing of 62 *UNG1* BIR outcomes, we observed that the outcomes accumulated a total of 41 G to N mutations on the right arm of chromosome III (Figure 5B). This is comparatively fewer than the 345 C to N mutations observed in the same chromosome region in the same 62 outcomes (Figure 5A, B), consistent with the higher frequency of A3A-induced Ura^+^ reversions in the Ori2 reporter than in the Ori1 reporter of our *UNG1* strains (Figure 4B). In the *ung1Δ* strains, C to N mutations accumulated massively on the BIR track with 2596 mutations across all 45 outcomes (Figure 5B). This accumulation of C to N mutations is in accordance with the idea that the majority of A3A-inflicted lesions (dU) are introduced in the nascent strand (lagging strand template) and that most of these lesions are channeled into an error-free pathway of repair by conversion into AP sites mediated by Ung1, as we previously proposed in (21). By contrast, we observed only 41 G to N mutations on the right arm of chromosome III in the *ung1Δ* background among all 45 outcomes analyzed (Figure 5B). We next compared the number of G to A mutations on the BIR track per outcome between *UNG1* (mean of 0.21 mutations per outcome) and *ung1Δ* (mean of 0.82 mutations per outcome) backgrounds and found that loss of *UNG1* promoted a significant increase (Figure 5C). In addition, when we compared the number of G to A mutations (mean of 0.21 per outcome) and G to C mutations (mean of 0.35 per outcome) among *UNG1* outcomes, there was no significant difference between the two, supporting that dU were efficiently converted into AP sites at least at some of the chromosomal positions (Figure 5D).

**Figure 6.**
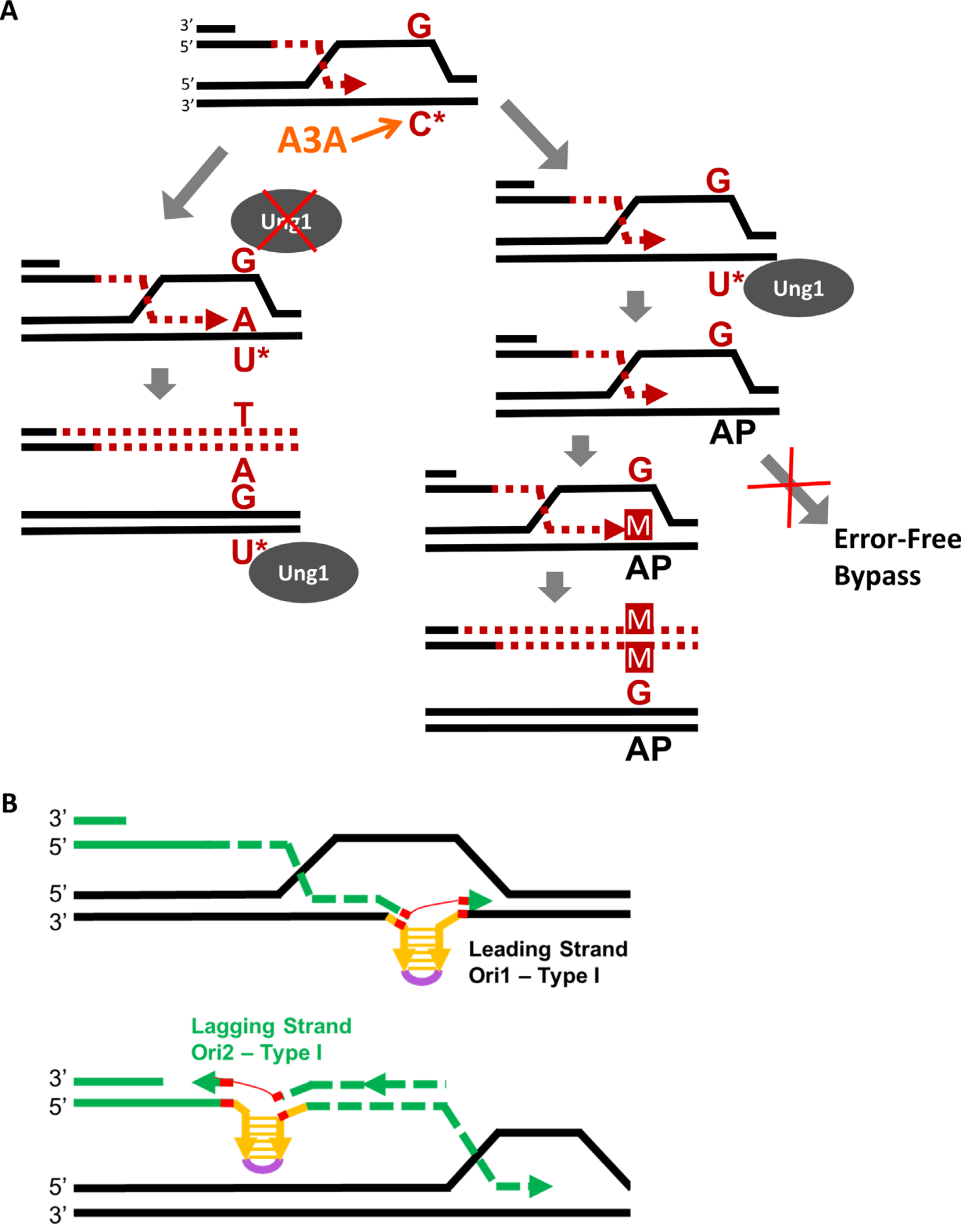
(**A**) Cytidine (C*) in the ssDNA of D-BTS is susceptible to A3A-induced deamination producing dU lesions (U*). The fate of dU lesions follows one of two possible paths. In the first (left), the Ung1 enzyme is unable to excise the dU base, leaving it in the template where it is encountered by leading strand synthesis. An A base is placed across from the dU lesion, incorporating it into the nascent strand where it is paired with a T base during lagging strand synthesis. Ung1 may later repair the template strand dU lesion after BIR has proceeded beyond it, but the mutation will stay in the newly synthesized strand. In the second path (right), Ung1 is able to access the dU lesion created by A3A and excise it, leaving an AP-site. This AP-site does not (or rarely) triggers error-free bypass pathways. Instead, a base placed across from the AP site during leading-strand synthesis often results in incorporation of the wrong base into the nascent strand, resulting in a mutation (M). (**B**) Schematics of Type I InsH deletion following hairpin formation during leading or lagging strand synthesis of BIR. Directions of leading and lagging strand synthesis are defined by the known direction of BIR synthesis for BIR in our system. Microhomologies at InsH deletion breakpoints are indicated in red.

>Notably, G to N and C to N mutations were nearly absent on the left arm of Chr. III in the *UNG1* background, but both were present in the *ung1Δ* background (Figure 5A), indicating that spontaneous dUs responsible for their formation did not frequently lead to mutations in the presence of uracil glycosylase. As a secondary control, we also identified C to N and G to N mutations across the rest of the genome (where BIR synthesis did not take place) in our sequenced outcomes in both *UNG1* and *ung1Δ* backgrounds. Particularly in the *UNG1* background, these mutations did not occur at the same frequency as those found on the BIR track, which represents only about 1% of the yeast genome (41 mutations observed on the BIR track vs 98 G to N mutations observed across the entirety of the genome (Supplementary Figure S4A; Supplementary Data S7)). Further, we performed simulations to test whether the number of G to N mutations that occur cumulatively across all *UNG1* background samples subjected to WGS were likely to occur in the observed frequency at which they exist on the BIR track region of the genome if redistributed randomly across the entire genome (Supplementary Figure 4B). From 100 000 simulations, we saw that most instances had 1–2 G to N mutations on the BIR track and the probability approached 0 between 7 and 8 G to N mutations. This was far fewer than the 41 G to N mutations that we observed in this region among all sequenced *UNG1* samples (Supplementary Figure 4B), thus strongly suggesting that these 41 G to N mutations that we report here indeed result from BIR. In addition, we confirmed that the majority of mutations detected in the *UNG1* and *ung1Δ* outcomes were part of a TCW motif, a recognition motif for A3A (Supplemental Figure 4C), as expected. From these results, we conclude that the ssDNA of the BIR D-loop is susceptible to A3A-induced damage with each lesion resulting in mutation more frequently than similar lesion introduced into ssDNA of the newly synthesized leading strand (Figure 6, see Discussion).

## DISCUSSION

Our results demonstrate that ssDNA formed in the D-BTS region is sufficient to form secondary structures and to incur DNA damage from agents that specifically target persistent ssDNA. This conclusion is based on our observations suggesting that the template for leading strand BIR synthesis is highly vulnerable to A3A damage and also highly susceptible to microhomology-mediated deletions promoted by inverted DNA repeats.

### The template for leading strand BIR synthesis is a novel source of mutagenic ssDNA

APOBEC-induced mutagenesis has been an important tool for identifying sources of persistent ssDNA *in vivo*. Previously, vulnerability to APOBEC-induced mutagenesis helped to identify several important sources of mutagenic ssDNA, including the lagging strand of S-phase DNA synthesis, actively transcribed regions (i.e. tRNA transcription), as well as uncapped telomeres (11,13–15,20,22,24,25,63). Our previous work on BIR (4,21) demonstrated that large amounts of stable nascent ssDNA accumulate during BIR leading strand DNA synthesis and following DSB resection preceding BIR. We observed that this ssDNA is susceptible to A3A-inflicted lesions and to alkylating damage, both leading to formation of mutations along the track of BIR that are similar to those termed *kataegis* that were described in cancer cells (4,5,16). While the majority of mutagenic ssDNA associated with BIR accumulates as the template for lagging strand synthesis, our new data presented here provide the evidence for mutagenic ssDNA formed in the template for the leading strand as well. This mutagenic DNA is formed in shorter stretches (creating isolated mutations rather than mutation clusters) that correspond to the template regions that become single-stranded within a D-loop. This conclusion is based on our analysis of mutation frequencies using the *ura3-29* reporter inserted at 16kb and 90kb positions on the BIR track. It is also supported by the results of our WGS analysis that identified mutations that likely resulted from dUs introduced in the D-BTS through the entire track of BIR.

Our conclusion regarding ssDNA in D-BTS is consistent with the previously published *in vitro* re-constitution of Polδ-driven repair DNA synthesis (87), suggesting that binding of RPA occurs within the D-loop to the template ssDNA and stimulates Polδ-driven repair DNA synthesis. The length of such ssDNA-binding RPA was proposed to be at least 30bp (required for binding one RPA molecule). The results of our study suggest that the length of this ssDNA may be much longer (at least 130-150 bp), because deletions of InsH, which provide an estimate of the size of the hairpin structure formed, were usually longer than 120bp. In addition, the results of our *in vivo* study allowed us to conclude that the ssDNA region within a D-loop not only exists but is likely persistent enough to become a significant source of mutagenesis. It is also possible that ssDNA in the D-BTS arises most readily when BIR proceeds slowly or interrupts. Nonetheless, our observation that D-BTS-associated mutations occur through the entire track of BIR suggests that instances of mutagenic ssDNA in the D-BTS arise often, regardless of whether perturbed BIR synthesis is prerequisite.

Previous studies demonstrated that correction of dUs introduced during replication or transcription usually (in ∼95% of the cases) proceeds via error-free repair (20,63). That was also the case for dU lesions introduced by APOBEC in the nascent strand of BIR (21). Here we demonstrate that dUs formed in the D-BTS are less frequently repaired in an error-free fashion and therefore lead to mutations more often. This could be caused by lower uracil glycosylase efficiency in excising of dUs or by lower efficiency of error-free repair pathways (template switching, homologous recombination, etc.) recruited for the repair of AP lesions. Variations in the efficiency of dU correction by Ung1 between different sources of ssDNA have been previously reported. For example, dUs introduced by APOBEC into yeast uncapped telomeres or into the nascent strand of BIR were almost all converted into AP sites by Ung1 as evidenced by the equal numbers of C to T and C to G base substitutions among repair outcomes and by increase in mutation frequency following elimination of Ung1 (11,21,63). Conversely, excision of dUs formed during S-phase replication (primarily in the lagging strand ssDNA) by Ung1 was ∼9% less efficient as compared to dUs excised during repair synthesis at uncapped telomeres (63). Our data reported here suggest that decreased efficiency of dU excision contributes to the increased level of mutations resulting from dUs in the D-BTS at the 16kb *ura3-29* position. However, because G to A and G to C base substitutions were observed in equal numbers through the track of BIR, it appears that Ung1 is capable of excising dUs at many BIR track locations. More striking, however, is the lack (or only modest increase) of G to N mutation frequencies in *ung1Δ* as compared to *UNG1* through the entire track of BIR. This data suggests that even when dUs in the D-BTS are converted into AP sites, error-free repair of these AP sites is not as efficient as was observed for the nascent BIR strand (Figure 6A versus (21)) or for S-phase lagging strand synthesis (63). Together, we propose that the transient state of ssDNA in the D-BTS region does not provide enough time for conversion of dUs into AP sites or for the channeling of AP sites into error-free repair pathways. An alternative possibility is that it might be difficult for Ung1 or for proteins participating in error-free repair to access their substrates inside of the D-loop, and likewise in the D-BTS region.

Although mutations resulting from D-BTS lesions rarely formed even small clusters, varying densities of G to A mutations along the entire track of BIR might be indicative of fluctuating lengths and persistence of ssDNA formed inside the D-BTS during BIR. Additional work, particularly in situations where D-loop migration is challenged by obstacles that lead to stalling, may elucidate factors capable of altering the amount of ssDNA present or amount of time that it persists within the D-loop, thereby increasing its propensity to accumulate damage, mutations, and mutation clusters. Another important observation from our WGS results was the presence of template strand A3A lesions upstream (centromere-proximal) to the HO cut site. Previously, A3A-induced mutations identified in the region between the centromere and the HO site were ascribed to long resection of DSB ends preceding strand invasion and BIR initiation (4,21). However, all mutations resulting from resection should occur at cytosines, not guanines. Yet, we identified several G to N mutations bearing A3A’s preferential sequence motif on the proposed resection track. We interpret this as an indication of synthesis in this region that leads to formation of these mutations caused by ssDNA lesions inside the D-BTS. This implies that damage to ssDNA exposed by long resection might not always be converted into mutations because the entire ssDNA region is removed if strand invasion occurs centromere-proximal to them. In this case, A3A-induced mutations likely result from synthesis initiated distant from the DSB following extensive resection.

### The ssDNA in the D-BTS promotes deletions at microhomologies stimulated by inverted DNA repeats

Here we report that BIR stimulates deletions that are promoted by inverted repeats and occur between microhomologies showing polarity (with one short repeat located inside of the IR and the other outside (see Figures 2E, G, 6B). Similar polarity was previously reported for IR-mediated deletions observed in *LYS2* genes during S-phase replication (36). To explain their mechanism, authors proposed that deletions are initiated by hairpin formation involving IRs included into ssDNA regions formed during lagging strand DNA synthesis. Further, it was proposed that displacement DNA synthesis driven by Polδ enters the duplex of the hairpin stem, but is unstable, which leads to frequent template switches from microhomology located inside of the hairpin duplex towards microhomology located outside of the hairpin (36). Based on this model, the polarity of deletion breakpoints allowed authors to postulate the direction of replication that led to deletions. Using the same logic, we use the polarity of insH deletion breakpoints that we observed during BIR (Figures 2B, E, G) to deduce the direction of synthesis producing the deletions. The result of our analysis indicates that both leading and lagging strand BIR synthesis promote IR-mediated deletions. This conclusion can be made based on Type I deletion frequencies following BIR in strains containing InsH in Ori1 and Ori2 orientations. Type I deletions were ∼5-fold more frequent in Ori2 (where they likely occur during lagging strand synthesis) as compared to Ori1 (leading strand synthesis) (Figures 2D, 6B). This difference is expected as ssDNA in the template for lagging strand BIR synthesis is expected to be longer and more stable as compared to the template for leading strand. Yet, deletions during leading strand synthesis were hundreds of times more frequent as compared to frequencies observed during S-phase DNA synthesis (Figure 1B, 2D, no DSB versus 7 h DSB). Similarly, the contribution of both leading and lagging strand BIR synthesis to the formation of IR-promoted deletions follows from the spectra of deletions observed by deep sequencing following BIR in Ori1 and Ori2 strains. In particular, after BIR, deletions of both polarities were observed (Type I-like (“left” microhomology inside the IR and the “right” microhomology outside—shown in red in Figures 2B, E, G, and 6B) and Type II-like (“right” microhomology inside the IR the “left” microhomology outside—shown in blue). The presence of both polarities following BIR in each strain is consistent with contributions of both leading and lagging strands to the induction of IR-promoted deletions.

Because Pol δ mediates both leading and lagging strand synthesis during BIR (77), and imprecise deletions were the predominant outcomes from both leading and lagging BIR synthesis, it is likely that both cases result from template switching occurring during displacement synthesis by Pol δ that partially opens hairpin stems and then undergoes slippage and switches to microhomology outside of the hairpin. We did not observe any evidence of translesion polymerase participation in the process, which also supports this model where slippage events are entirely mediated by Pol δ. The effect of the *pol3-t* mutation in this context (including the increase in the overall frequency of InsH deletions, and in the fraction of precise deletions among them) might result from reduced displacement ability of Pol δ in the *pol3-t* mutant leading to more frequent stalling of Pol δ at the base of hairpins. An additional explanation for the high frequency of imprecise deletions observed during BIR is that they result from the formation of incomplete hairpins due to a limited amount of ssDNA exposed during BIR. This could explain the effects of *pol3-t* on events attributed to leading strand synthesis as this mutant might increase the length of ssDNA in the D-BTS. However, this cannot explain why BIR lagging strand synthesis does not solely produce precise deletions when the amount of ssDNA accumulated after leading strand synthesis should be sufficient for full hairpin formation, even in *POL3* (wt) strains. In addition, our failure to recapitulate the effect of *pol3-t* in *POL3* (wild-type) cells by executing BIR at a lower temperature to slow down BIR synthesis also makes the second explanation less likely, even though it is possible that the decreased temperature slows down not only DNA synthesis but also DNA unwinding. Overall, the first explanation (Pol δ-mediated displacement synthesis as an explanation for imprecise deletions of InsH) appears more likely, even though the latter explanation could represent another contributing factor. It is also possible that ssDNA accumulated in the D-BTS region is less accessible for RPA binding, which could represent an additional factor provoking more efficient hairpin formation. Finally, questions remain about whether the genetic requirements for deletions in *pol3-t* are different from those in *POL3*. For example, we cannot exclude that deletions of InsH in *pol3-t* could be mediated by another polymerase (for example by Polζ).

### BIR-associated mutagenic ssDNA: future questions

The observations made in this study prompted us to formulate several additional questions.

First, our findings led us to wonder whether D-BTS regions form during other homologous recombination pathways (e.g., during gene conversion (GC)) and are also mutagenic. Because the D-BTS is likely a common feature of GC and BIR, the mutagenic properties of the D-BTS could be shared between these two pathways. It would be especially relevant to investigate this with respect to meiotic recombination events because many D-loops are formed during every meiosis and because mutagenesis associated with meiosis could have severe consequences for progeny, potentially leading to birth defects in humans.

The results obtained in this work might also help in the interpreting of the mechanisms responsible for the formation of APOBEC-induced mutation clusters that are detected in various cancers. For example, it was believed that only non-switching C-coordinated or G-coordinated clusters can be ascribed to BIR (1,17). It is clear from this study that mutagenesis from both leading and lagging BIR strands is expected to produce more complex patterns, such as clusters where most of the mutations are G- or C-coordinated with rare cases of mutations in the opposing base (e.g. rare mutation in G in otherwise C-coordinated clusters and *visa versa*), similar to what was observed in (17).

## DATA AVAILABILITY

Raw sequencing reads can be accessed from the NCBI Sequence Read Archive database under bioproject accession number PRJNA821991. In addition, the raw reads that were re-analyzed in this work and were originally from (21) can be accessed from NCBI Sequence Read Archive database under accession number PRJNA517571. All custom code used for the analysis is available through GitHub https://github.com/malkovalab/WGS-A3A-Tools; (https://github.com/malkovalab/DeepSeqTools).

## Supplementary Material

gkac520_Supplemental_Files

## References

[1] Saini N. , Gordenin D.A. Hypermutation in single-stranded DNA. DNA Repair (Amst). 2020; 91-92:102868. 32438271 10.1016/j.dnarep.2020.102868 PMC7234795

[2] Chatterjee N. , Walker G.C. Mechanisms of DNA damage, repair, and mutagenesis. Environ. Mol. Mutagen. 2017; 58:235–263. 28485537 10.1002/em.22087 PMC5474181

[3] Chan K. , Resnick M.A., Gordenin D.A. The choice of nucleotide inserted opposite abasic sites formed within chromosomal DNA reveals the polymerase activities participating in translesion DNA synthesis. DNA Repair (Amst). 2013; 12:878–889. 23988736 10.1016/j.dnarep.2013.07.008 PMC3825800

[4] Sakofsky C.J. , Roberts S.A., Malc E., Mieczkowski P.A., Resnick M.A., Gordenin D.A., Malkova A. Break-induced replication is a source of mutation clusters underlying *Kataegis*. Cell Reports. 2014; 7:1640–1648. 24882007 10.1016/j.celrep.2014.04.053 PMC4274036

[5] Roberts S.A. , Sterling J., Thompson C., Harris S., Mav D., Shah R., Klimczak L.J., Kryukov G.V., Malc E., Mieczkowski P.A. Clustered mutations in yeast and in human cancers can arise from damaged long single-strand DNA regions. Molecular cell. 2012; 46:424–435. 22607975 10.1016/j.molcel.2012.03.030 PMC3361558

[6] Degtyareva N.P. , Heyburn L., Sterling J., Resnick M.A., Gordenin D.A., Doetsch P.W. Oxidative stress-induced mutagenesis in single-strand DNA occurs primarily at cytosines and is DNA polymerase zeta-dependent only for adenines and guanines. Nucleic Acids Res. 2013; 41:8995–9005. 23925127 10.1093/nar/gkt671 PMC3799438

[7] Degtyareva N.P. , Saini N., Sterling J.F., Placentra V.C., Klimczak L.J., Gordenin D.A., Doetsch P.W. Mutational signatures of redox stress in yeast single-strand DNA and of aging in human mitochondrial DNA share a common feature. PLoS Biol. 2019; 17:e3000263. 31067233 10.1371/journal.pbio.3000263 PMC6527239

[8] Roberts S.A. , Gordenin D.A. Clustered and genome-wide transient mutagenesis in human cancers: Hypermutation without permanent mutators or loss of fitness. Bioessays. 2014; 36:382–393. 24615916 10.1002/bies.201300140 PMC4145046

[9] Harris R.S. Molecular mechanism and clinical impact of APOBEC3B-catalyzed mutagenesis in breast cancer. Breast Cancer Res. 2015; 17:8. 25848704 10.1186/s13058-014-0498-3 PMC4303225

[10] Swanton C. , McGranahan N., Starrett G.J., Harris R.S. APOBEC enzymes: mutagenic fuel for cancer evolution and heterogeneity. Cancer Discov. 2015; 5:704–712. 26091828 10.1158/2159-8290.CD-15-0344 PMC4497973

[11] Chan K. , Sterling J.F., Roberts S.A., Bhagwat A.S., Resnick M.A., Gordenin D.A. Base damage within single-strand DNA underlies in vivo hypermutability induced by a ubiquitous environmental agent. PLoS Genet. 2012; 8:e1003149. 23271983 10.1371/journal.pgen.1003149 PMC3521656

[12] Taylor B.J. , Nik-Zainal S., Wu Y.L., Stebbings L.A., Raine K., Campbell P.J., Rada C., Stratton M.R., Neuberger M.S. DNA deaminases induce break-associated mutation showers with implication of APOBEC3B and 3A in breast cancer *kataegis*. Elife. 2013; 2:e00534. 23599896 10.7554/eLife.00534 PMC3628087

[13] Bhagwat A.S. , Hao W., Townes J.P., Lee H., Tang H., Foster P.L. Strand-biased cytosine deamination at the replication fork causes cytosine to thymine mutations in Escherichia coli. Proc. Natl. Acad. Sci. U.S.A. 2016; 113:2176–2181. 26839411 10.1073/pnas.1522325113 PMC4776466

[14] Lada A.G. , Dhar A., Boissy R.J., Hirano M., Rubel A.A., Rogozin I.B., Pavlov Y.I. AID/APOBEC cytosine deaminase induces genome-wide kataegis. Biol. Direct. 2012; 7:47. 23249472 10.1186/1745-6150-7-47 PMC3542020

[15] Lada A.G. , Stepchenkova E.I., Waisertreiger I.S., Noskov V.N., Dhar A., Eudy J.D., Boissy R.J., Hirano M., Rogozin I.B., Pavlov Y.I. Genome-wide mutation avalanches induced in diploid yeast cells by a base analog or an APOBEC deaminase. PLoS Genet. 2013; 9:e1003736. 24039593 10.1371/journal.pgen.1003736 PMC3764175

[16] Nik-Zainal S. , Alexandrov L.B., Wedge D.C., Van Loo P., Greenman C.D., Raine K., Jones D., Hinton J., Marshall J., Stebbings L.A. et al. Mutational processes molding the genomes of 21 breast cancers. Cell. 2012; 149:979–993. 22608084 10.1016/j.cell.2012.04.024 PMC3414841

[17] Sakofsky C.J. , Saini N., Klimczak L.J., Chan K., Malc E.P., Mieczkowski P.A., Burkholder A.B., Fargo D., Gordenin D.A. Repair of multiple simultaneous double-strand breaks causes bursts of genome-wide clustered hypermutation. PLoS Biol. 2019; 17:e3000464. 31568516 10.1371/journal.pbio.3000464 PMC6786661

[18] Roberts S.A. , Lawrence M.S., Klimczak L.J., Grimm S.A., Fargo D., Stojanov P., Kiezun A., Kryukov G.V., Carter S.L., Saksena G. et al. An APOBEC cytidine deaminase mutagenesis pattern is widespread in human cancers. Nat. Genet. 2013; 45:970–976. 23852170 10.1038/ng.2702 PMC3789062

[19] Burch L.H. , Yang Y., Sterling J.F., Roberts S.A., Chao F.G., Xu H., Zhang L., Walsh J., Resnick M.A., Mieczkowski P.A. et al. Damage-induced localized hypermutability. Cell Cycle. 2011; 10:1073–1085. 21406975 10.4161/cc.10.7.15319 PMC3100884

[20] Saini N. , Roberts S.A., Sterling J.F., Malc E.P., Mieczkowski P.A., Gordenin D.A. APOBEC3B cytidine deaminase targets the non-transcribed strand of tRNA genes in yeast. DNA Repair (Amst). 2017; 53:4–14. 28351647 10.1016/j.dnarep.2017.03.003 PMC5450012

[21] Elango R. , Osia B., Harcy V., Malc E., Mieczkowski P.A., Roberts S.A., Malkova A. Repair of base damage within break-induced replication intermediates promotes kataegis associated with chromosome rearrangements. Nucleic Acids Res. 2019; 47:9666–9684. 31392335 10.1093/nar/gkz651 PMC6765108

[22] Hoopes J.I. , Cortez L.M., Mertz T.M., Malc E.P., Mieczkowski P.A., Roberts S.A. APOBEC3A and APOBEC3B preferentially deaminate the lagging strand template during DNA replication. Cell Rep. 2016; 14:1273–1282. 26832400 10.1016/j.celrep.2016.01.021 PMC4758883

[23] Chan K. , Gordenin D.A. Clusters of Multiple Mutations: Incidence and Molecular Mechanisms. Annu. Rev. Genet. 2015; 49:243–267. 26631512 10.1146/annurev-genet-112414-054714 PMC4710516

[24] Chan K. , Roberts S.A., Klimczak L.J., Sterling J.F., Saini N., Malc E.P., Kim J., Kwiatkowski D.J., Fargo D.C., Mieczkowski P.A. et al. An APOBEC3A hypermutation signature is distinguishable from the signature of background mutagenesis by APOBEC3B in human cancers. Nat. Genet. 2015; 47:1067–1072. 26258849 10.1038/ng.3378 PMC4594173

[25] Sui Y. , Qi L., Zhang K., Saini N., Klimczak L.J., Sakofsky C.J., Gordenin D.A., Petes T.D., Zheng D.Q. Analysis of APOBEC-induced mutations in yeast strains with low levels of replicative DNA polymerases. Proc. Natl. Acad. Sci. U.S.A. 2020; 117:9440–9450. 32277034 10.1073/pnas.1922472117 PMC7196835

[26] Lobachev K.S. , Rattray A., Narayanan V. Hairpin- and cruciform-mediated chromosome breakage: causes and consequences in eukaryotic cells. Front. Biosci. 2007; 12:4208–4220. 17485368 10.2741/2381

[27] Bissler J.J. DNA inverted repeats and human disease. Front. Biosci. 1998; 3:D408–D418. 9516381 10.2741/a284

[28] Tanaka H. , Bergstrom D.A., Yao M.C., Tapscott S.J. Widespread and nonrandom distribution of DNA palindromes in cancer cells provides a structural platform for subsequent gene amplification. Nat. Genet. 2005; 37:320–327. 15711546 10.1038/ng1515

[29] Neil A.J. , Kim J.C., Mirkin S.M. Precarious maintenance of simple DNA repeats in eukaryotes. Bioessays. 2017; 39:10.1002/bies.201700077. PMC5577815 28703879

[30] Khristich A.N. , Armenia J.F., Matera R.M., Kolchinski A.A., Mirkin S.M. Large-scale contractions of Friedreich's ataxia GAA repeats in yeast occur during DNA replication due to their triplex-forming ability. Proc. Natl. Acad. Sci. U.S.A. 2020; 117:1628–1637. 31911468 10.1073/pnas.1913416117 PMC6983365

[31] van Wietmarschen N. , Merzouk S., Halsema N., Spierings D.C., Guryev V., Lansdorp P.M. BLM helicase suppresses recombination at G-quadruplex motifs in transcribed genes. Nat. Commun. 2018; 9:271. 29348659 10.1038/s41467-017-02760-1 PMC5773480

[32] Bacolla A. , Tainer J.A., Vasquez K.M., Cooper D.N. Translocation and deletion breakpoints in cancer genomes are associated with potential non-B DNA-forming sequences. Nucleic Acids Res. 2016; 44:5673–5688. 27084947 10.1093/nar/gkw261 PMC4937311

[33] Tanaka H. , Yao M.C. Palindromic gene amplification–an evolutionarily conserved role for DNA inverted repeats in the genome. Nat. Rev. Cancer. 2009; 9:216–224. 19212324 10.1038/nrc2591

[34] Tran H.T. , Degtyareva N.P., Koloteva N.N., Sugino A., Masumoto H., Gordenin D.A., Resnick M.A. Replication slippage between distant short repeats in Saccharomyces cerevisiae depends on the direction of replication and the *RAD50* and *RAD52* genes. Mol. Cell. Biol. 1995; 15:5607–5617. 7565712 10.1128/mcb.15.10.5607 PMC230811

[35] Lobachev K.S. , Shor B.M., Tran H.T., Taylor W., Keen J.D., Resnick M.A., Gordenin D.A. Factors affecting inverted repeat stimulation of recombination and deletion in *Saccharomyces cerevisiae*. Genetics. 1998; 148:1507–1524. 9560370 10.1093/genetics/148.4.1507 PMC1460095

[36] Gordenin D. , Lobachev K., Degtyareva N., Malkova A., Perkins E., Resnick M. Inverted DNA repeats: a source of eukaryotic genomic instability. Mol. Cell. Biol. 1993; 13:5315–5322. 8395002 10.1128/mcb.13.9.5315-5322.1993 PMC360228

[37] Gordenin D. , Malkova A., Peterzen A., Kulikov V., Pavlov Y., Perkins E., Resnick M. Transposon Tn5 excision in yeast: influence of DNA polymerases alpha, delta, and epsilon and repair genes. Proc. Natl. Acad. Sci. U.S.A. 1992; 89:3785–3789. 1315039 10.1073/pnas.89.9.3785 PMC525575

[38] Lemoine F.J. , Degtyareva N.P., Kokoska R.J., Petes T.D. Reduced levels of DNA polymerase delta induce chromosome fragile site instability in yeast. Mol Cell Biol. 2008; 28:5359–5368. 18591249 10.1128/MCB.02084-07 PMC2519721

[39] Lemoine F.J. , Degtyareva N.P., Lobachev K., Petes T.D. Chromosomal translocations in yeast induced by low levels of DNA polymerase a model for chromosome fragile sites. Cell. 2005; 120:587–598. 15766523 10.1016/j.cell.2004.12.039

[40] VanHulle K. , Lemoine F.J., Narayanan V., Downing B., Hull K., McCullough C., Bellinger M., Lobachev K., Petes T.D., Malkova A. Inverted DNA repeats channel repair of distant double-strand breaks into chromatid fusions and chromosomal rearrangements. Mol. Cell. Biol. 2007; 27:2601–2614. 17242181 10.1128/MCB.01740-06 PMC1899885

[41] Rattray A.J. , Shafer B.K., Neelam B., Strathern J.N. A mechanism of palindromic gene amplification in *Saccharomyces cerevisiae*. Genes Dev. 2005; 19:1390–1399. 15937224 10.1101/gad.1315805 PMC1142561

[42] Narayanan V. , Mieczkowski P.A., Kim H.M., Petes T.D., Lobachev K.S. The pattern of gene amplification is determined by the chromosomal location of hairpin-capped breaks. Cell. 2006; 125:1283–1296. 16814715 10.1016/j.cell.2006.04.042

[43] Carvalho C.M. , Lupski J.R. Mechanisms underlying structural variant formation in genomic disorders. Nat. Rev. Genet. 2016; 17:224–238. 26924765 10.1038/nrg.2015.25 PMC4827625

[44] Osia B. , Alsulaiman T., Jackson T., Kramara J., Oliveira S., Malkova A. Cancer cells are highly susceptible to accumulation of templated insertions linked to MMBIR. Nucleic Acids Res. 2021; 49:8714–8731. 34379776 10.1093/nar/gkab685 PMC8421209

[45] Lobachev K.S. , Gordenin D.A., Resnick M.A. The Mre11 complex is required for repair of hairpin-capped double-strand breaks and prevention of chromosome rearrangements. Cell. 2002; 108:183–193. 11832209 10.1016/s0092-8674(02)00614-1

[46] Malkova A. , Haber J.E. Mutations arising during repair of chromosome breaks. Annu. Rev. Genet. 2012; 46:455–473. 23146099 10.1146/annurev-genet-110711-155547

[47] Ponder R.G. , Fonville N.C., Rosenberg S.M. A switch from high-fidelity to error-prone DNA double-strand break repair underlies stress-induced mutation. Mol. Cell. 2005; 19:791–804. 16168374 10.1016/j.molcel.2005.07.025

[48] Holbeck S.L. , Strathern J.N. A role for *REV3* in mutagenesis during double-strand break repair in *Saccharomyces cerevisiae*. Genetics. 1997; 147:1017–1024. 9383049 10.1093/genetics/147.3.1017 PMC1208230

[49] McGill C.B. , Shafer B.K., Derr L.K., Strathern J.N. Recombination initiated by double-strand breaks. Curr. Genet. 1993; 23:305–314. 8467528 10.1007/BF00310891

[50] Yang Y. , Sterling J., Storici F., Resnick M.A., Gordenin D.A. Hypermutability of damaged single-strand DNA formed at double-strand breaks and uncapped telomeres in yeast *Saccharomyces cerevisiae*. PLoS Genet. 2008; 4:e1000264. 19023402 10.1371/journal.pgen.1000264 PMC2577886

[51] Saini N. , Ramakrishnan S., Elango R., Ayyar S., Zhang Y., Deem A., Ira G., Haber J.E., Lobachev K.S., Malkova A. Migrating bubble during break-induced replication drives conservative DNA synthesis. Nature. 2013; 502:389–392. 24025772 10.1038/nature12584 PMC3804423

[52] Kramara J. , Osia B., Malkova A. Break-induced replication: the where, the why, and the how. Trends Genet. 2018; 34:518–531. 29735283 10.1016/j.tig.2018.04.002 PMC6469874

[53] Anand R.P. , Lovett S.T., Haber J.E. Break-induced DNA replication. Cold Spring Harb. Perspect. Biol. 2013; 5:a010397. 23881940 10.1101/cshperspect.a010397 PMC3839615

[54] Llorente B. , Smith C.E., Symington L.S. Break-induced replication: what is it and what is it for?. Cell Cycle. 2008; 7:859–864. 18414031 10.4161/cc.7.7.5613

[55] Sakofsky C.J. , Malkova A. Break induced replication in eukaryotes: mechanisms, functions, and consequences. Crit Rev. Biochem. Mol. Biol. 2017; 52:395–413. 28427283 10.1080/10409238.2017.1314444 PMC6763318

[56] Davis A.P. , Symington L.S. RAD51-dependent break-induced replication in yeast. Mol. Cell. Biol. 2004; 24:2344–2351. 14993274 10.1128/MCB.24.6.2344-2351.2004 PMC355873

[57] Lydeard J.R. , Jain S., Yamaguchi M., Haber J.E. Break-induced replication and telomerase-independent telomere maintenance require Pol32. Nature. 2007; 448:820–823. 17671506 10.1038/nature06047

[58] Malkova A. , Naylor M.L., Yamaguchi M., Ira G., Haber J.E. RAD51-dependent break-induced replication differs in kinetics and checkpoint responses from RAD51-mediated gene conversion. Mol. Cell. Biol. 2005; 25:933–944. 15657422 10.1128/MCB.25.3.933-944.2005 PMC544012

[59] Ruff P. , Donnianni R., Glancy E., Oh J., Symington L. RPA Stabilization of Single-Stranded DNA Is Critical for Break-Induced Replication. Cell Rep. 2016; 17:3359–3368. 28009302 10.1016/j.celrep.2016.12.003 PMC5218512

[60] Chung W.H. , Zhu Z., Papusha A., Malkova A., Ira G. Defective Resection at DNA Double-Strand Breaks Leads to *De Novo* Telomere Formation and Enhances Gene Targeting. PLoS Genet. 2010; 17:3359–3368. 10.1371/journal.pgen.1000948 PMC2869328 20485519

[61] Wilson M.A. , Kwon Y., Xu Y., Chung W.H., Chi P., Niu H., Mayle R., Chen X., Malkova A., Sung P. et al. Pif1 helicase and Poldelta promote recombination-coupled DNA synthesis via bubble migration. Nature. 2013; 502:393–396. 24025768 10.1038/nature12585 PMC3915060

[62] Donnianni R.A. , Symington L.S. Break-induced replication occurs by conservative DNA synthesis. Proc. Natl. Acad. Sci. U.S.A. 2013; 110:13475–13480. 23898170 10.1073/pnas.1309800110 PMC3746906

[63] Hoopes J.I. , Hughes A.L., Hobson L.A., Cortez L.M., Brown A.J., Roberts S.A. Avoidance of APOBEC3B-induced mutation by error-free lesion bypass. Nucleic Acids Res. 2017; 45:5243–5254. 28334887 10.1093/nar/gkx169 PMC5605239

[64] Deem A. , Barker K., Vanhulle K., Downing B., Vayl A., Malkova A. Defective break-induced replication leads to half-crossovers in *Saccharomyces cerevisiae*. Genetics. 2008; 179:1845–1860. 18689895 10.1534/genetics.108.087940 PMC2516063

[65] Deem A. , Keszthelyi A., Blackgrove T., Vayl A., Coffey B., Mathur R., Chabes A., Malkova A. Break-induced replication is highly inaccurate. PLoS Biol. 2011; 9:e1000594. 21347245 10.1371/journal.pbio.1000594 PMC3039667

[66] Gordenin D. , Trofimova M., Shaburova O., Pavlov Y.I., Chernoff Y.O., Chekuolene Y., Proscyavichus Y., Sasnauskas K., Janulaitis A. Precise excision of bacterial transposon Tn 5 in yeast. Mol. Gen. Genet. 1988; 213:388–393. 2847007 10.1007/BF00339607

[67] Storici F. , Resnick M.A. *Delitto perfetto* targeted mutagenesis in yeast with oligonucleotides. Genet Eng (N Y). 2003; 25:189–207. 15260239

[68] Storici F. , Resnick M.A. The delitto perfetto approach to in vivo site-directed mutagenesis and chromosome rearrangements with synthetic oligonucleotides in yeast. Methods Enzymol. 2006; 409:329–345. 16793410 10.1016/S0076-6879(05)09019-1

[69] Wach A. , Brachat A., Pöhlmann R., Philippsen P. New heterologous modules for classical or PCR-based gene disruptions in *Saccharomyces cerevisiae*. Yeast. 1994; 10:1793–1808. 7747518 10.1002/yea.320101310

[70] Gueldener U. , Heinisch J., Koehler G., Voss D., Hegemann J. A second set of loxP marker cassettes for Cre-mediated multiple gene knockouts in budding yeast. Nucleic Acids Res. 2002; 30:e23. 11884642 10.1093/nar/30.6.e23 PMC101367

[71] Pavlov Y.I. , Shcherbakova P.V., Kunkel T.A. In vivo consequences of putative active site mutations in yeast DNA polymerases α, ϵ, δ, and ζ. Genetics. 2001; 159:47–64. 11560886 10.1093/genetics/159.1.47 PMC1461793

[72] Kokoska R.J. , Stefanovic L., Tran H.T., Resnick M.A., Gordenin D.A., Petes T.D. Destabilization of yeast micro-and minisatellite DNA sequences by mutations affecting a nuclease involved in Okazaki fragment processing (rad27) and DNA polymerase δ (pol3-t). Mol. Cell. Biol. 1998; 18:2779–2788. 9566897 10.1128/mcb.18.5.2779 PMC110657

[73] Anand R. , Beach A., Li K., Haber J. Rad51-mediated double-strand break repair and mismatch correction of divergent substrates. Nature. 2017; 544:377–380. 28405019 10.1038/nature22046 PMC5544500

[74] Elango R. , Kockler Z., Liu L., Malkova A. Investigation of break-induced replication in yeast. Methods Enzymol. 2018; 601:161–203. 29523232 10.1016/bs.mie.2017.12.010

[75] Sakofsky C.J. , Ayyar S., Deem A.K., Chung W.H., Ira G., Malkova A. Translesion polymerases drive microhomology-mediated break-induced replication leading to complex chromosomal rearrangements. Mol. Cell. 2015; 60:860–872. 26669261 10.1016/j.molcel.2015.10.041 PMC4688117

[76] Osia B. , Elango R., Kramara J., Roberts S.A., Malkova A. Investigation of break-induced replication in yeast. Methods Mol. Biol. 2021; 2153:307–328. 32840789 10.1007/978-1-0716-0644-5_22 PMC9041317

[77] Donnianni R.A. , Zhou Z.-X., Lujan S.A., Al-Zain A., Garcia V., Glancy E., Burkholder A.B., Kunkel T.A., Symington L.S. DNA polymerase delta synthesizes both strands during break-induced replication. Mol. Cell. 2019; 76:371–381. 31495565 10.1016/j.molcel.2019.07.033 PMC6862718

[78] Pavlov Y.I. , Shcherbakova P.V., Rogozin I.B. Roles of DNA polymerases in replication, repair, and recombination in eukaryotes. Int Rev Cytol. 2006; 255:41–132. 17178465 10.1016/S0074-7696(06)55002-8

[79] Tran H.T. , Degtyareva N.P., Gordenin D.A., Resnick M.A. Genetic factors affecting the impact of DNA polymerase∂ proofreading activity on mutation avoidance in yeast. Genetics. 1999; 152:47–59. 10224242 10.1093/genetics/152.1.47 PMC1460598

[80] Northam M.R. , Robinson H.A., Kochenova O.V., Shcherbakova P.V. Participation of DNA polymerase zeta in replication of undamaged DNA in *Saccharomyces cerevisiae*. Genetics. 2010; 184:27–42. 19841096 10.1534/genetics.109.107482 PMC2815923

[81] Northam M.R. , Moore E.A., Mertz T.M., Binz S.K., Stith C.M., Stepchenkova E.I., Wendt K.L., Burgers P.M., Shcherbakova P.V. DNA polymerases zeta and Rev1 mediate error-prone bypass of non-B DNA structures. Nucleic Acids Res. 2014; 42:290–306. 24049079 10.1093/nar/gkt830 PMC3874155

[82] Vasan S. , Deem A., Ramakrishnan S., Argueso J.L., Malkova A. Cascades of genetic instability resulting from compromised break-induced replication. PLoS Genet. 2014; 10:e1004119. 24586181 10.1371/journal.pgen.1004119 PMC3937135

[83] Morrison A. , Johnson A.L., Johnston L.H., Sugino A. Pathway correcting DNA replication errors in *Saccharomyces cerevisiae*. EMBO J. 1993; 12:1467–1473. 8385605 10.1002/j.1460-2075.1993.tb05790.x PMC413358

[84] Hicks W.M. , Kim M., Haber J.E. Increased mutagenesis and unique mutation signature associated with mitotic gene conversion. Science. 2010; 329:82–85. 20595613 10.1126/science.1191125 PMC4254764

[85] Stith C.M. , Sterling J., Resnick M.A., Gordenin D.A., Burgers P.M. Flexibility of eukaryotic Okazaki fragment maturation through regulated strand displacement synthesis. J. Biol. Chem. 2008; 283:34129–34140. 18927077 10.1074/jbc.M806668200 PMC2590699

[86] Shcherbakova P.V. , Pavlov Y.I. 3'→5' exonucleases of DNA polymerases epsilon and delta correct base analog induced DNA replication errors on opposite DNA strands in *Saccharomyces cerevisiae*. Genetics. 1996; 142:717–726. 8849882 10.1093/genetics/142.3.717 PMC1207013

[87] Sneeden J.L. , Grossi S.M., Tappin I., Hurwitz J., Heyer W.D. Reconstitution of recombination-associated DNA synthesis with human proteins. Nucleic Acids Res. 2013; 41:4913–4925. 23535143 10.1093/nar/gkt192 PMC3643601

