# Supplementary material for: Migrating bubble synthesis promotes mutagenesis through lesions in its template
Source: Nucleic Acids Res. 2022 Jun 24;50(12):6870–89. doi: 10.1093/nar/gkac520 (PMC9262586; doi:10.1093/nar/gkac520)
Supplement: gkac520_Supplemental_Files [file gkac520_supplemental_files.zip › Supplementary information 404.pdf]

Supplementary Information

Migrating bubble synthesis promotes mutagenesis through lesions in its template

Beth Osia[1,2,#], Jerzy Twarowski[1#], Tyler Jackson[1,3], Liping Liu[1], and Anna Malkova[1*]

Supplementary Figure Legends

Supplementary Figure 1. Spectra of InsH deletions at various reporter positions. (**A**) Deletion spectra of strains harboring the *lys2-InsH* reporter construct at *MAT*, 16kb, and 36kb positions (as described in **Figure 1A**) The data for 16kb are the same as in Fig. 3D for *POL3*. (**B**) Transformation efficiencies (per 1ml of culture transformed with 1.5μg of DNA) measured by the frequency of Lys$^+$ colonies formed when cells are transformed with PCR-amplified wild-type *LYS2*, imperfect deletion *LYS2** alleles (Type I and Type II), or no DNA control (measuring spontaneous reversion when no template is provided) into strains containing a *lys2-A$_4$* allele (Lys$^-$) at 3 positions (*MAT*, 16kb, and 36kb). Median values are displayed above each bar and error bars indicate range (n=4 for each strain and DNA/no DNA combination).

Supplementary Figure 2. Quantification of BIR-associated Type I deletions of insH by ddPCR. The output of one ddPCR run, which included BIR samples (left 4 samples) obtained from strains containing *lys2-InsH* reporter at 16kb in two orientations (Ori1 and Ori2) and no-DSB controls (right four samples) is presented. Results of ddPCR for Type I deletion shown by 1D pictures from Quantasoft Software (Biorad). After completion of ddPCR, all droplets were analyzed by the QX200 ddPCR system and the concentration of template DNA was calculated by Poisson distribution based on the number of positive and negative droplets in each sample. The Y-axis of each 1D picture represents the fluorophore signal of each droplet generated during the PCR reaction. The threshold line (pink horizontal line) was drawn manually at the space between positive and negative (plus background) droplets.

Supplemental Figure 3. The effect of temperature of incubation during BIR on Lys$^+$ frequency. (**A**) The effect of low temperature (20˚C) during BIR as compared to *POL3* (30˚C) and effect of *pol3-t* (30˚C) as compared to *POL3* (30˚C) on Lys$^+$ reversion rates in strains harboring the *lys2-InsH* reporter at the 16kb position. Asterisk indicates significantly different value (P<0.05, actual P-value listed above) and N.S. indicates no significant difference (P ≥0.05). See Supplementary Data S5 for 95% CI of 20˚C experiments. Other details similar to **Figure 3A and B.** The data for *POL3* (30˚C) and for *pol3-t* (30˚C) are the same as in **Figure 3B.** (**B**) Deletion spectra of *lys2-InsH* in *POL3* and *pol3-t* strains. Temperature of incubation during BIR is indicated. P-values are listed to indicate statistically significant differences (P<0.05) measured by Fisher's exact test from *POL3* (30˚C) strains for the fractions of precise and Type I deletions. N.S. indicates no significant difference (P≥0.05). The data for *POL3* (30˚C) and *pol3-t* (30˚C) are the same as in **Figure 3D**.

Supplemental Figure 4. Analysis of BIR/APOBEC3A-associated mutagenesis by whole genome sequencing. (A) Summary of all C to N and G to N mutations found across the whole genomes of 62 *UNG1* BIR outcomes shown also in Figure 5A and Supplementary Data S7. Mutation types are as indicated in the legend. **(B)** Distribution of G to N mutations occurring on the BIR track if all observed G to N mutations (shown in A) were redistributed across the entire genome at random. Results obtained from 100000 simulated synthetic genomes (see Materials and Methods for details). Red vertical line represents the observed number of G to N mutations in the right arm of chromosome III. **(C)** DNA base frequency logos for mutated reference cytosines (C) and guanines (G) (center base) shown in *UNG1* outcomes 1-25 and *ung1Δ* outcomes 1-25 shown in Figure 5A and flanking 5 bases on either side. A3A TCW/WGA motif positions are indicated above each logo.

Supplementary Data.

Supplementary Data S1. Table of Yeast Strains

Supplementary Data S2. Lys^+ mutation rates for lys2-InsH reporter system wild-type and translesion polymerase mutants

Supplementary Data S3. Deep sequencing deletion event read counts and summary

Supplementary Data S4. *lys2-InsH* deletions detected by deep sequencing

Supplementary Data S5. Lys^+mutation rates calculated for *POL3* mutants

Supplementary Data S6. Ura^+mutation rates calculated for expression of A3A and Empty Vector

Supplementary Data S7. Filtered SNP Calls for WGS of strains containing *ura3-29* reporter at 90kb

A

	Precise Deletion	Type I Deletion	Type II Deletion
MAT	2/13<br>15%	11/13<br>85%	0/13<br>0%
16kb	1/43<br>2%	28/43<br>65%	14/43<br>33%
36kb	0/32<br>0%	32/32<br>100%	0/32<br>0%

B

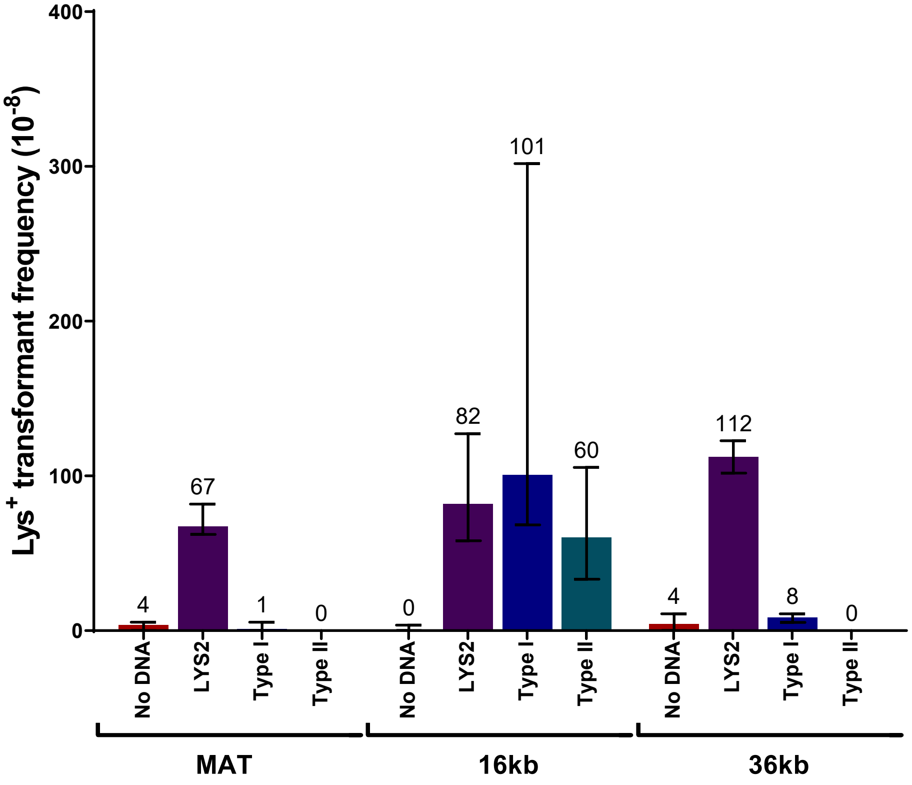

Osia et al, Supplemental Figure 2

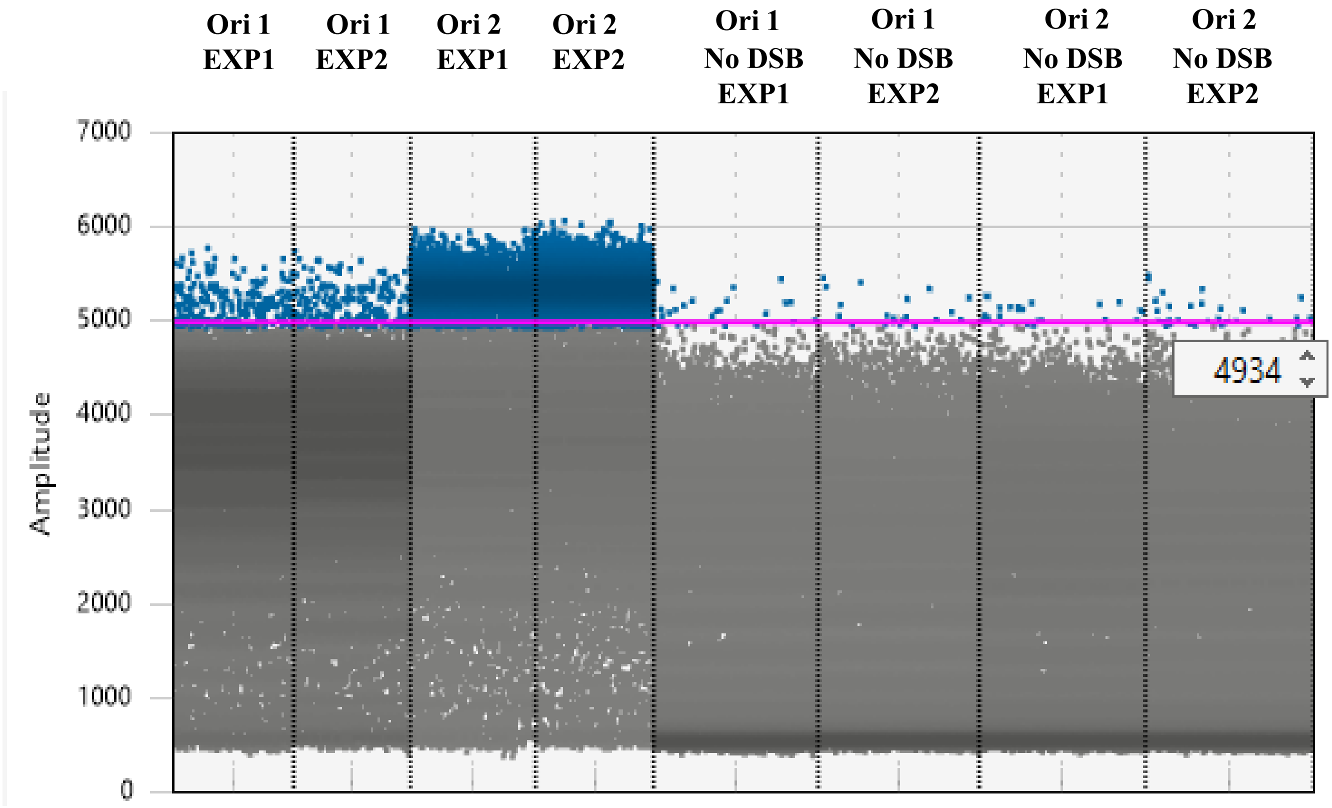

A

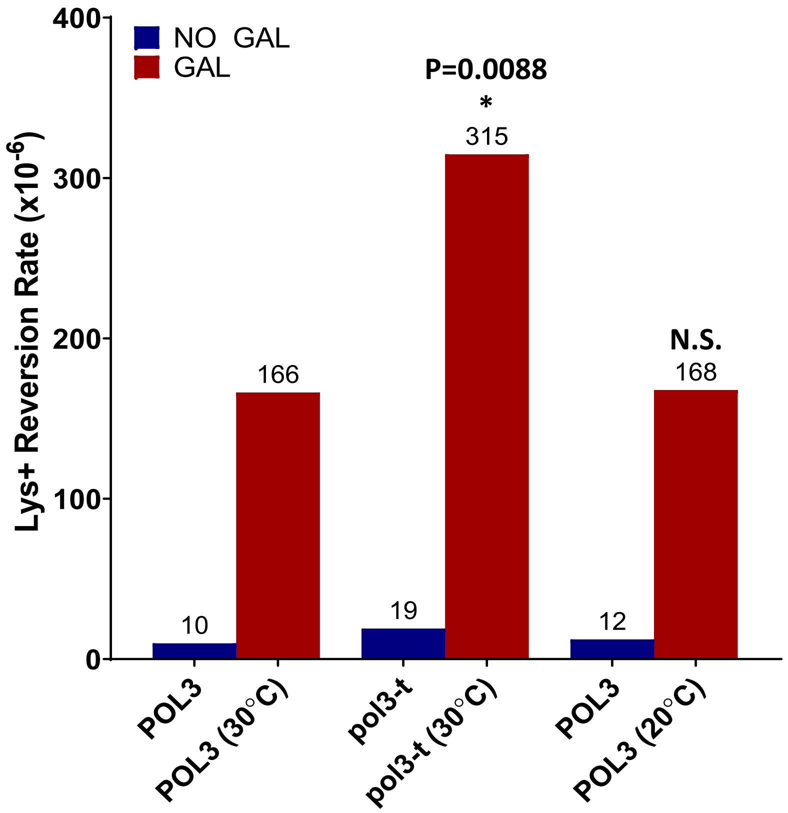

B

	Precise Deletion	Type I Deletion	Type II Deletion
**POL3** ***30*°C**	1/43<br>**2%**	28/43<br>**65%**	14/43<br>**33%**
pol3-t ***30*°C**	13/26<br>**50%**<br>P=0.0067	9/26<br>**35%**<br>P=0.0241	4/26<br>**15%**<br>N.S.
POL3 ***20*°C**	1/41<br>**2%**<br>N.S.	28/41<br>**68%**<br>N.S.	12/41<br>**29%**<br>N.S.

Osia et al, Supplemental Figure 4

A

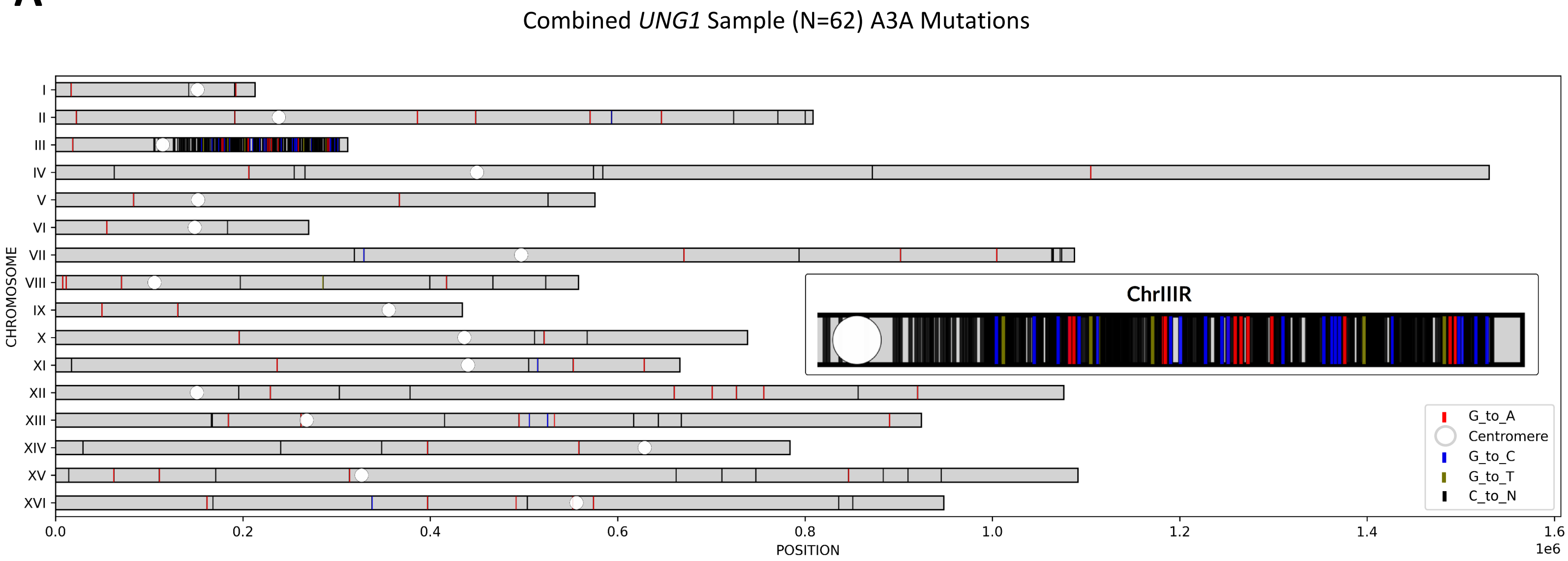

B

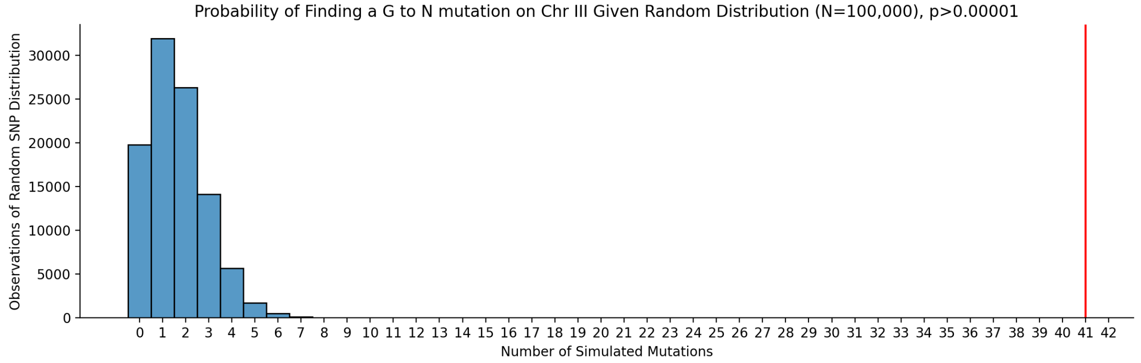

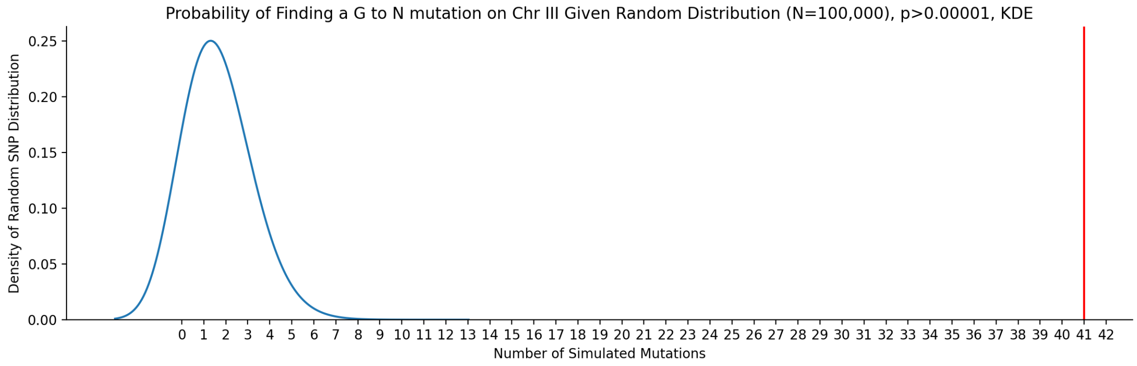

C

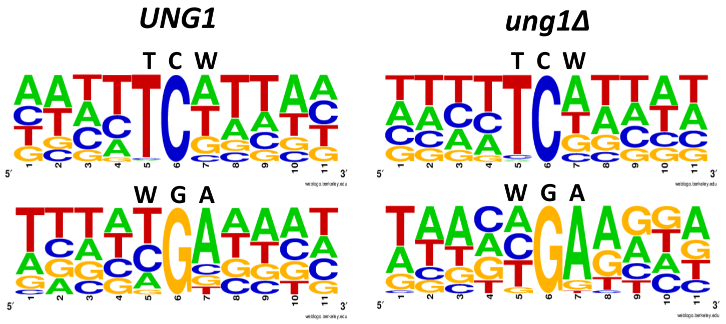

Supplementary Data S1.Table of Yeast Strains

AM1003	*hml*Δ*::ADE1/hml*Δ*::ADE3 MAT**a**-LEU2-tel/MAT*α*-inc hmr*Δ*::HPH FS2*Δ*::NAT/FS2 leu2/leu2-3,112 thr4 ura3-52 ade3::GAL::HO ade1 met13*	(64)
AM4466	*hml*Δ*::ADE1/hml*Δ*::ADE3 MAT**a**-LEU2-tel/MAT*α*-inc hmr*Δ*::HPH FS2*Δ*::NAT/FS2 leu2/leu2-3,112 ura3-52 ade3::GAL::HO ade1 met13 lys2Δ* and *lys2-InsH* (ori1) at 16kb position (Chr. III)	This Study
AM4947	*hml*Δ*::ADE1/hml*Δ*::ADE3 MAT**a**-LEU2-tel/MAT*α*-inc hmr*Δ*:: HPH FS2*Δ*::NAT/FS2 leu2/leu2-3,112 ura3-52 ade3::GAL::HO ade1 met13 lys2Δ* and *lys2-InsH* (ori2) at 16kb position (Chr. III)	This Study
AM4461	*hml*Δ*::ADE1/hml*Δ*::ADE3 MAT**a**-LEU2-tel/ MAT*α*-inc :: lys2-InsH hmr*Δ*::HPH FS2*Δ*::NAT/FS2 leu2/leu2-3,112 ura3-52 ade3::GAL::HO ade1 met13 lys2Δ*	This Study
AM4471	*hml*Δ*::ADE1/hml*Δ*::ADE3 MAT**a**-LEU2-tel/MAT*α*-inc hmr*Δ*::HPH FS2*Δ*::NAT/FS2 leu2/leu2-3,112 ura3-52 ade3::GAL::HO ade1 met13 lys2Δ* and *lys2-InsH* at 36kb position (Chr. III)	This Study

AM4607	AM4466, but *MAT**a**::MATα-inc* (No-DSB control).	This Study
AM6500	AM4947 but *MAT**a**::MATα-inc* (No-DSB control)	This Study
AM4605	AM4461, but *MAT**a**::MAT α-inc* (No-DSB control)	This Study
AM4611	AM4471, but *MAT**a**::MAT α-inc* (No-DSB control)	This Study
AM4718	AM4466, but *rev3::BSD*	This Study
AM4722	AM4466, but *rad30::KanMX*	This Study
AM4801	AM4466, but *HPH::Bleo^r pol3-01*	This Study
AM4849	AM4466, but *rev3::BSD rad30::KanMX*	This Study
AM5032	AM4466, but *pol3-t*	This Study
AM5088	AM4466, but *pol3-Y708A*	This Study
AM6504	AM4466, but *pol4:: KanMX/ pol4:: KanMX/*	This Study
AM5089	AM4466, but *rev3::BSD pol3-Y708A*	This Study
AM1411	AM1003, but *THR4 lys2Δ MATα-inc::lys2-InsA$_4$*	(65)
AM1291	AM1003, but *lys2Δ, thr4::lys2-InsA$_4$*	(65)
AM4329	AM1003, but *THR4 lys2Δ, lys2-InsA$_4$* at 36kb-position	This Study

AM5594	*hml*Δ*::ADE1/hml*Δ*::ADE3 MAT**a**-LEU2-tel/MATα-inc hmr*Δ*:: KanMX FS2*Δ*::NAT/FS2 leu2/leu2-3,112 ura3-52 ade3::GAL::HO ade1 met13 lys2Δ ura3Δ thr4::ura3-29::Bleo*^{r} (ori1, 16-kb-position)	This Study
AM5770	AM5594, but *ung1::BSD*	This Study
AM3529	*hml*Δ*::ADE1/hml*Δ*::ADE3 MAT**a**-LEU2-tel/MATα-inc hmr*Δ*:: KanMX FS2*Δ*::NAT/FS2 leu2/leu2-3,112 ura3-52 ade3::GAL::HO ade1 met13 lys2Δ ura3Δ thr4::ura3-29::Bleo*^{r} (ori2, 16-kb-position)	This Study
AM5926	AM3529, but *ung1::BSD*	This Study
AM5373	*hml*Δ*::ADE1/hml*Δ*::ADE3 MAT**a**-LEU2-tel/MATα-inc hmr*Δ*:: Bleo*^{r} *FS2*Δ*::NAT/FS2 leu2/leu2-3,112 ura3-52 ade3::GAL::HO ade1 met13 lys2Δ ura3Δ ura3-29::Bleo*^{r} (ori1) at 90-kb-position)	This Study
AM6218	*AM5373, but ung1::KANMX*	This Study

Supplementary Data S4. *lys2-InsH* deletions detected by deep sequencing

Deletion type annotation [a]	Deletion class [b]	Deletion type [c]	Number of excised bases [d]
(No Deletion)	(No Deletion)	lys2_consensus	NA
...GTTAGGACGTTCTCCAAAGAACTACAGTTTCAAAGTGTTTGCCCACGTCAGGGCCTGACTCTTATACACAAGTAGCGTCCTGAACGGAACCTTTCCCGTTTTCCAGGATCTGATCTTCCATGTTAGGAGGTCACATGGAAGATCAGATCCTGGAAAACGGGAAAGGTTCCGTTCAGGACGCTACTTGTGTATAAGAGTCAGCGTCAGGGCCAAGGATGAAGAAGCTGCATTTGCAAGATTACAAAAGGCAGGTATCACCTATGGTACTTGGAACGAAAAATTTGCCTCAAATATT...			
1.	Type-II-like	type2	171
...GTT**AGGACG**CTACTTGTGTATAAGAGTCAGCGTCAGGGCCAAGGATGAAGAAGCTGCATTTGCAAGATTACAAAAGGCAGGTATCACCTATGGTACTTGGAACGAAAAATTTGCCTCAAATATT...			
2.	Type-II-like	J7	130
...GTTAGGACGTTCTCCAAAGAACTACAGTTTC**AAAG**GTTCCGTTCAGGACGCTACTTGTGTATAAGAGTCAGCGTCAGGGCCAAGGATGAAGAAGCTGCATTTGCAAGATTACAAAAGGCAGGTATCACCTATGGTACTTGGAACGAAAAATTTGCCTCAAATATT...			
3.	Type-II-like	J2	152
...GTTAGGACGTTCTCCAAAGAACTACAGTTTCAAA**GTGT**ATAAGAGTCAGCGTCAGGGCCAAGGATGAAGAAGCTGCATTTGCAAGATTACAAAAGGCAGGTATCACCTATGGTACTTGGAACGAAAAATTTGCCTCAAATATT...			
4.	Type-II-like	J6	126
...GTTAGGACGTTCTCCAAAGAACTACAGTTTCAAAGTGTTTGC**CC**GTTCAGGACGCTACTTGTGTATAAGAGTCAGCGTCAGGGCCAAGGATGAAGAAGCTGCATTTGCAAGATTACAAAAGGCAGGTATCACCTATGGTACTTGGAACGAAAAATTTGCCTCAAATATT...			
5.	Type-II-like	type2_v1	95
...GTTAGGACGTTCTCCAAAGAACTACAGTTTCAAAGTGTTTGCCCACG**TCAG**ATCCTGGAAAACGGGAAAGGTTCCGTTCAGGACGCTACTTGTGTATAAGAGTCAGCGTCAGGGCCAAGGATGAAGAAGCTGCATTTGCAAGATTACAAAAGGCAGGTATCACCTATGGTACTTGGAACGAAAAATTTGCCTCAAATATT...			
6.	Type-II-like	type2_v2	150
...GTTAGGACGTTCTCCAAAGAACTACAGTTTCAAAGTGTTTGCCCAC**GTCAG**CGTCAGGGCCAAGGATGAAGAAGCTGCATTTGCAAGATTACAAAAGGCAGGTATCACCTATGGTACTTGGAACGAAAAATTTGCCTCAAATATT...			
7.	Type-II-like	J1	125
...GTTAGGACGTTCTCCAAAGAACTACAGTTTCAAAGTGTTTGCCCACG**TCAGG**ACGCTACTTGTGTATAAGAGTCAGCGTCAGGGCCAAGGATGAAGAAGCTGCATTTGCAAGATTACAAAAGGCAGGTATCACCTATGGTACTTGGAACGAAAAATTTGCCTCAAATATT...			
8.	Symmetrical	full_deletion	156
...GTTAGGACGTTCTCCAAAGAACTACAGTTTCAAAGTGTTTGCCCA**CGTCAGGGC**CAAGGATGAAGAAGCTGCATTTGCAAGATTACAAAAGGCAGGTATCACCTATGGTACTTGGAACGAAAAATTTGCCTCAAATATT...			
9.	Type-I-like	type1	126
...GTTAGGACGTTCTCCAAAGAACTACAGTTTCAAAGTGTTTGCCCACGTCAGGGCCTGACTCTTATACACAAGT**AGCGTC**AGGGCCAAGGATGAAGAAGCTGCATTTGCAAGATTACAAAAGGCAGGTATCACCTATGGTACTTGGAACGAAAAATTTGCCTCAAATATT...			
10.	Type-I-like	J5	189
...GTTAGGACGTTCTCCAAAGAACTACAGTTTCAAAGTGTTTGCCCACGTCAGGGCCTGACTCTTATACACAAGTAGCGTCCT**GAACG**AAAAATTTGCCTCAAATATT...			
11.	Type-I-like	J4	189
...GTTAGGACGTTCTCCAAAGAACTACAGTTTCAAAGTGTTTGCCCACGTCAGGGCCTGACTCTTATACACAAGTAGCGTCCTGAACGGAAAA**TTT**GCCTCAAATATT...			
12.	Type-I-like	J3	189

...GTTAGGACGTTCTCCAAAGAACTACAGTTTCAAAGTGTTTGCCCACGTCAGGGCCTGACTCTTATACACAAGTAGCGTCCTGAACGGAACCTTTC**CC**TCAAATATT...			
13.	Type-I-like	type5	109
...GTTAGGACGTTCTCCAAAGAACTACAGTTTCAAAGTGTTTGCCCACGTCAGGGCCTGACTCTTATACACAAGTAGCGTCCTGAACGGAACCTTTCCCGTTTTCCA**GGAT**AAGAAGCTGCATTTGCAAGATTACAAAAGGCAGGTATCACCTATGGTACTTGGAACGAAAAATTTGCCTCAAATATT...			
Unnumbered 1.	Other	flipped_spacer	NA
...GTTAGGACGTTCTCCAAAGAACTACAGTTTCAAAGTGTTTGCCCACGTCAGGGCCTGACTCTTATACACAAGTAGCGTCCTGAACGGAACCTTTCCCGTTTTCCAGGATCTGATCTTCCATGT**GACCTCCTA**ACATGGAAGATCAGATCCTGGAAAACGGGAAAGGTTCCGTTCAGGACGCTACTTGTGTATAAGAGTCAGCGTCAGGGCCAAGGATGAAGAAGCTGCATTTGCAAGATTACAAAAGGCAGGTATCACCTATGGTACTTGGAACGAAAAATTTGCCTCAAATATT...			
Unnumbered 2.	Other	internal_deletion	70
...GTTAGGACGTTCTCCAAAGAACTACAGTTTCAAAGTGTTTGCCCACGTCAGGGCCTGACTCTTATACACAAGTAGCG**TCCTG**GAAAACGGGAAAGGTTCCGTTCAGGACGCTACTTGTGTATAAGAGTCAGCGTCAGGGCCAAGGATGAAGAAGCTGCATTTGCAAGATTACAAAAGGCAGGTATCACCTATGGTACTTGGAACGAAAAATTTGCCTCAAATATT...			

[a] A reference number used for each deletion type in Figure 2E.

[b] Deletion Class as identified through the position of deletion events. Deletion event types with parts of the 5' side of the hairpin structure preserved were classified as "Type-I-like", and events with parts of the 3' side of the hairpin structure preserved were classified as "Type-II-like". Full hairpin deletions using Tn5 direct repeat were classified as "Symmetrical". Other identifiable event types, that were neither full deletions, "Type-I-like" or "Type-II-like", were classified as "Other". Reads that didn't support any of the known deletion event types were labeled as "Unclassified".

[c] Name of the individual deletion event types with identical homologies (bold and underlined sequence fragments) used for the fragment deletion.

[d] Number of total bases deleted in the product from the *lys2-InsH* reporter.
